# *Annurca* apple polyphenol extract promotes mesenchymal-to-epithelial transition and inhibits migration in triple-negative breast cancer cells through ROS/JNK signaling

**DOI:** 10.1038/s41598-020-73092-2

**Published:** 2020-09-28

**Authors:** Daniela Cristina Vuoso, Stefania D’Angelo, Rosalia Ferraro, Sergio Caserta, Stefano Guido, Marcella Cammarota, Marina Porcelli, Giovanna Cacciapuoti

**Affiliations:** 1 Department of Precision Medicine, University of Campania “Luigi Vanvitelli”, Via Luigi De Crecchio 7, 80138 Naples, Italy; 2 Department of Motor Sciences and Wellness, “Parthenope” University, Via Medina 40, 80133 Naples, Italy; 3 Department of Chemical, Materials and Industrial Production Engineering (DICMAPI), University of Naples Federico II, P.le Tecchio 80, 80125 Naples, Italy; 4 CEINGE Advanced Biotechnologies, 80145 Naples, Italy; 5 Department of Experimental Medicine, University of Campania “Luigi Vanvitelli”, Via Luciano Armanni 5, 80138 Naples, Italy

**Keywords:** Biochemistry, Oncology

## Abstract

Aberrant activation of epithelial-to-mesenchymal transition has been shown to correlate with triple-negative breast cancer (TNBC) progression and metastasis. Thus, the induction of the reverse process might offer promising opportunities to restrain TNBC metastatic spreading and related mortality. Recently, the *Annurca* apple polyphenol extract (APE) has been highlighted as a multi-faceted agent that selectively kills TNBC cells by ROS generation and sustained JNK activation. Here, by qualitatively and quantitatively monitoring the real-time movements of live cells we provided the first evidence that APE inhibited the migration of MDA-MB-231 and MDA-MB-468 TNBC cells and downregulated metalloproteinase-2 and metalloproteinase-9. In MDA-MB-231 cells APE decreased SMAD-2/3 and p-SMAD-2/3 levels, increased E-cadherin/N-cadherin protein ratio, induced the switch from N-cadherin to E-cadherin expression and greatly reduced vimentin levels. Confocal and scanning electron microscopy imaging of APE-treated MDA-MB-231 cells evidenced a significant cytoskeletal vimentin and filamentous actin reorganization and revealed considerable changes in cell morphology highlighting an evident transition from the mesenchymal to epithelial phenotype with decreased migratory features. Notably, all these events were reverted by *N*-acetyl-l-cysteine and JNK inhibitor SP600125 furnishing evidence that APE exerted its effects through the activation of ROS/JNK signaling. The overall data highlighted APE as a potential preventing agent for TNBC metastasis.

## Introduction

Breast cancer is the most common invasive cancer among women in the world. Although over the past two decades treatment for breast cancer has substantially improved, its metastasis is still a major cause of mortality and poor prognosis^1^. Migration and invasion, during which cancer cells move through the primary tumor and reach the blood vessels, are regarded as a crucial step for initial breast cancer metastasis^2^. Thus, identifying chemical compounds targeting cancer cell migration and invasion is highly desirable.

Conversion of early-to-advanced stage tumors is associated with activation of epithelial-mesenchymal transition (EMT), a process playing a crucial role in driving carcinoma invasion and metastasis^3^. EMT is a reversible multistep and highly regulated trans-differentiation process where polarized epithelial cells lose the so-called epithelial markers and undergo changes in morphology and cytoskeletal organization, acquiring motile mesenchymal traits. EMT is also related to an acquired ability by tumor cells to evade senescence, apoptosis, and anoikis, which are needed for tumor dissemination and metastasis^2–4^.

EMT reprogramming of gene expression can be induced and modulated by signaling pathways that respond to a variety of extracellular signals among which transforming growth factor-beta (TGF-β) plays a predominant role^5^. The changes in gene expression from epithelial to mesenchymal phenotype are triggered by complex regulatory networks also involving a transcriptional control responsible for the repression of E-cadherin expression thus inducing and starting EMT process^2–4,6^.

Triple-negative breast cancer (TNBC) is a highly aggressive and invasive subgroup of breast carcinomas that lacks expression of estrogen and progesterone receptor and human epidermal growth factor receptor-2^7,8^. Proven active targeted therapy is presently unavailable for TNBC and currently chemotherapy represents the standard of care for patients with early advanced TNBC. Since EMT process would enable the invasive properties of TNBC, the induction of the reverse process mesenchymal-epithelial transition (MET), might offer new promising opportunities to restrain the metastatic spreading of TNBC and the related mortality.

Dietary polyphenols are among naturally-occurring substances that have shown hopeful anti-cancer properties and low toxicity in comparison to standard chemotherapeutic agents^9^.

Although most of the beneficial effects of natural polyphenols can be ascribed to their antioxidant properties, in cancer cells polyphenols may also have pro-oxidant activity and could act as selective cytotoxic agents through increasing reactive oxygen species (ROS) levels beyond critical threshold limits^10^. Accumulating evidence indicates that excessive ROS levels can induce cancer cell death by modulating c-Jun-*N*-terminal kinase (JNK), a stress-associated protein kinase belonging to mitogen-activated protein kinase family^11^ thus suggesting that targeting ROS/JNK signaling pathway might represent an effective strategy for cancer treatment.

*Annurca* apples, a southern Italian variety, are characterized by an extremely high content of polyphenols and were proved endowed with nutraceutical potential in many human conditions. The hundreds of different metabolites contained in *Annurca* apple polyphenol extract (APE) act in synergism and allow this extract to be effective in a plethora of different biological contexts: as an antioxidant, as a modulator of lipid and cholesterol anabolism, as hair growth promoter or against stress and aging^12,13^.

Previous works from our group led us to select APE as a promising nutraceutical approach to add on therapy against breast cancer. Indeed, we have reported that APE displayed a potent prooxidant cytotoxic effect in MCF-7 human breast carcinoma cells^14^ and more recently we demonstrated that APE was able to selectively kill MDA-MB-231 TNBC cells while exerting a protective antioxidant effect on MCF10A, a non-tumorigenic human mammary epithelial cell line^15^. Moreover, we furnished evidence that ROS are important mediators of cytotoxic effect exerted by APE in MDA-MB-231 cells and that JNK represents a crucial player downstream of ROS^15^. Herein, to deepen knowledge on APE anticancer effects, we investigated for the first time its potential in inhibiting the in vitro migration of MDA-MB-231 and MDA-MB-468 TNBC cells. Moreover, by monitoring specific morphological and biomolecular markers we highlighted the ability of APE to induce MET in MDA-MB-231 cells, enabling them to acquire a less invasive phenotype. Finally, we demonstrated that inhibition of cell migration and induction of MET by APE are mainly mediated by the activation of ROS/JNK signaling cascade. The present study provides the first evidence for APE as a potential antimetastatic agent for the treatment of highly invasive TNBC.

## Results

### Time-lapse video microscopy revealed in real-time APE-induced inhibition of TNBC cell migration

**Figure 1:**
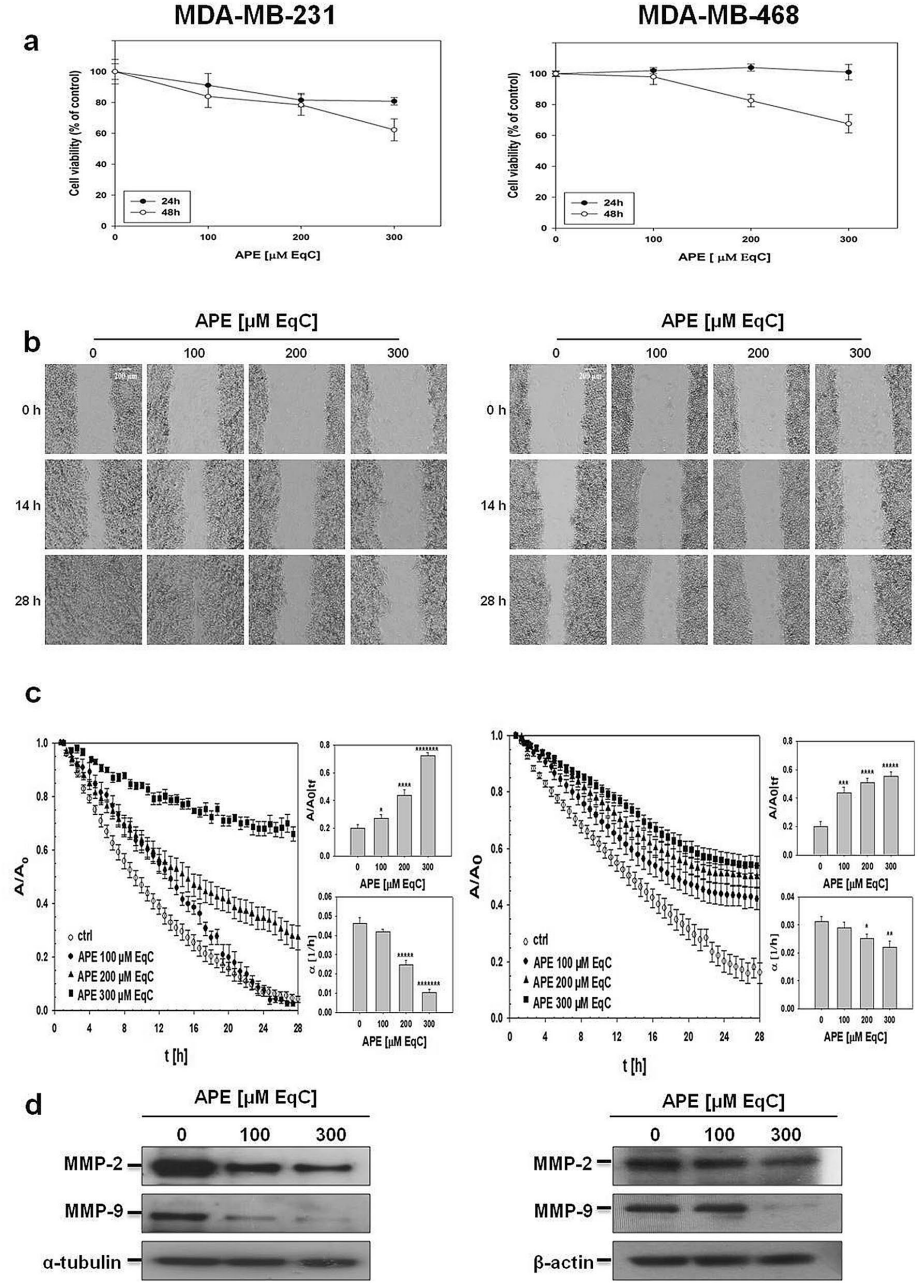
APE inhibited cell growth and migration of MDA-MB-231 and MDA-MB-468 cells. (**a**) MDA-MB-231 (left) and MDA-MB-468 (right) cells were cultured for 24 and 48 h in medium supplemented or not with APE at the indicated concentrations. Cell viability was then assessed by MTT assay and expressed as a percentage of untreated cells. Values represent the mean ± SD of three independent experiments. (**b**) Representative phase-contrast microscopy images showing the wound closure process at three different time points in MDA-MB-231 (left) and MDA-MB-468 (right) cells incubated or not with APE at the indicated concentrations. Images in the panels are relative to a single field of view, taken as qualitatively representative of a given experimental condition. Representative time-lapse videos related to MDA-MB-231 (videos S1–S4) and MDA-MB-468 (videos S5–S8) cells are included in Supplementary Information. (**c**) Evolution in time of the wound area A, normalized to the value A_0_ at time 0, for MDA-MB-231 (left) and MDA-MB-468 cells (right) incubated or not (ctrl) with APE. The linear range of each data series was fit in order to measure the wound closure velocity α. Upper bar diagram shows the values of the wound area A normalized to the value A_0_ for treated cells at time t_f_, when the A/A_0_ value for control is 0.2. Lower bar diagram shows the values of α (h^-1^) calculated at time t_f_ for the control and treated cells. The values reported in the histograms represent the mean from several independent fields of view. Standard error of the mean was calculated and one-tailed and heteroscedastic t-test were computed to verify the statistical significance of the differences with respect to control samples (*P < 0.05, **P < 1 × 10^–3^, ***P < 5 × 10^–4^, ****P < 5 × 10^–5^, *****P < 5 × 10^–6^, *******P < 5 × 10^–8^). (**d**) The levels of MMP-2 and MMP-9 in MDA-MB-231 (left) and MDA-MB-468 (right) cells treated with APE at the indicated concentrations for 48 h were measured by Western blot. All results were obtained from at least three independent experiments. α-tubulin and β-actin were used as standard for the equal loading of protein in the lanes. The full-length blots are included in Supplementary Information (Fig. S3).

The effect of APE on TNBC cell migration was investigated in mesenchymal-like MDA-MB-231, highly aggressive and invasive, and basal-like MDA-MB-468, characterized by a less invasive phenotype and metastatic potential^16^. Firstly, to assess APE cytotoxicity, cell viability was detected by MTT assay after treatment with increasing APE concentrations for 24 and 48 h. As shown in Fig. 1a, APE, after 24 h and at the highest concentration, caused only poor effect on MDA-MB-231 cells, while no cytotoxicity was observed in MDA-MB-468 cells. Thus, a 24 h incubation was selected to ensure that at least 80% of cells were viable during cell migration experiments.

Cell migration was monitored in real-time using video time-lapse microscopy. Representative phase-contrast microscopy images of the wound closure process as a function of time are shown in Fig. 1b for MDA-MB-231 (left) and MDA-MB-468 (right) cells incubated or not with increasing APE concentrations. Live-cell imaging showed, in both cell lines, a qualitatively higher closure of the wound in control respect to treated samples indicating that APE inhibited TNBC cells motility. Time-lapse videos of the wound healing of MDA-MB-231 (videos S1–S4) and MDA-MB-468 (videos S5–S8) cells are available as Supplementary Information. The wound closure dynamics for MDA-MB-231 (left) and MDA-MB-468 (right) cells in the presence and absence of APE were quantified by plotting the values of the wound area normalized to its value at time zero (A/A_0_) as a function of time (Fig. 1c). A typical curve was graphically analyzed in Fig. S1. The results indicated that in both cell lines control cells closed the wound region faster than treated cells and that APE slowed-down the wound closure in a dose-dependent manner. A further method for measuring the differences among treated and untreated samples was to calculate from raw data in Fig. 1c the A/A_0_ and the wound closure velocity (α) values at the time t_f_, (i.e. when A/A_0_ for control is 0.2). It has to be noted that t_f_ value for MDA-MB-231 cells is smaller than that of MDA-MB-468 probably due to their lower duplication time (38 h and 47 h, respectively). We observed that in both cell lines ${\left.\frac{A}{{A}_{0}}\right|}_{{t}_{f}}$ increased with the concentration of APE, reaching at 300 μM catechin equivalent (EqC) a value about fourfold and threefold higher than control for MDA-MB-231 and MDA-MB-468, respectively (Fig. 1c, upper bar diagrams). On the contrary, the wound healing velocity decreased as the concentration of APE in the culture medium raised (Fig. 1c, lower bar diagrams). Analysis of doubling and migration time allowed to evaluate the contribution of cell motility and proliferation to wound healing process (Supplementary Information) and the comparison of migration times of treated and untreated cells further confirmed the dose-dependent inhibitory effect of APE (Fig. S2).

### APE downregulated MMP-2 and MMP-9 in MDA-MB-231 and MDA-MB-468 cells

Degradation of the basement membrane and extracellular matrix (ECM) is carried out by various proteolytic enzymes, including matrix metalloproteinases (MMPs)^17^. To further explore the inhibitory effects of APE on tumor cell migration, the expression of MMP-2 and MMP-9 was detected by Western blot (Fig. 1d). As expected, due to their crucial role in the invasion and metastasis of malignant cells, after 48 h treatment with APE 100 μM and 300 μM EqC the levels of both enzymes decreased in a dose-dependent manner compared to control cells. Interestingly, the effect of APE was more pronounced in MDA-MB-231 cells and more remarkable on MMP-9, in line with literature data reporting that MMP-9 is strongly associated with aggressive and metastatic breast cancer^18^ and that MMP-9 drives malignant progression and metastasis of basal-like TNBC^19^. Altogether, these data indicated that the APE-inhibitory effect on MDA-MB-231 and MDA-MB-468 cell migration was associated with MMP-2 and MMP-9 downregulation.

### APE decreased mesenchymal and increased epithelial markers expression and downregulated SMAD signaling in MDA-MB-231 cells

**Figure 2:**
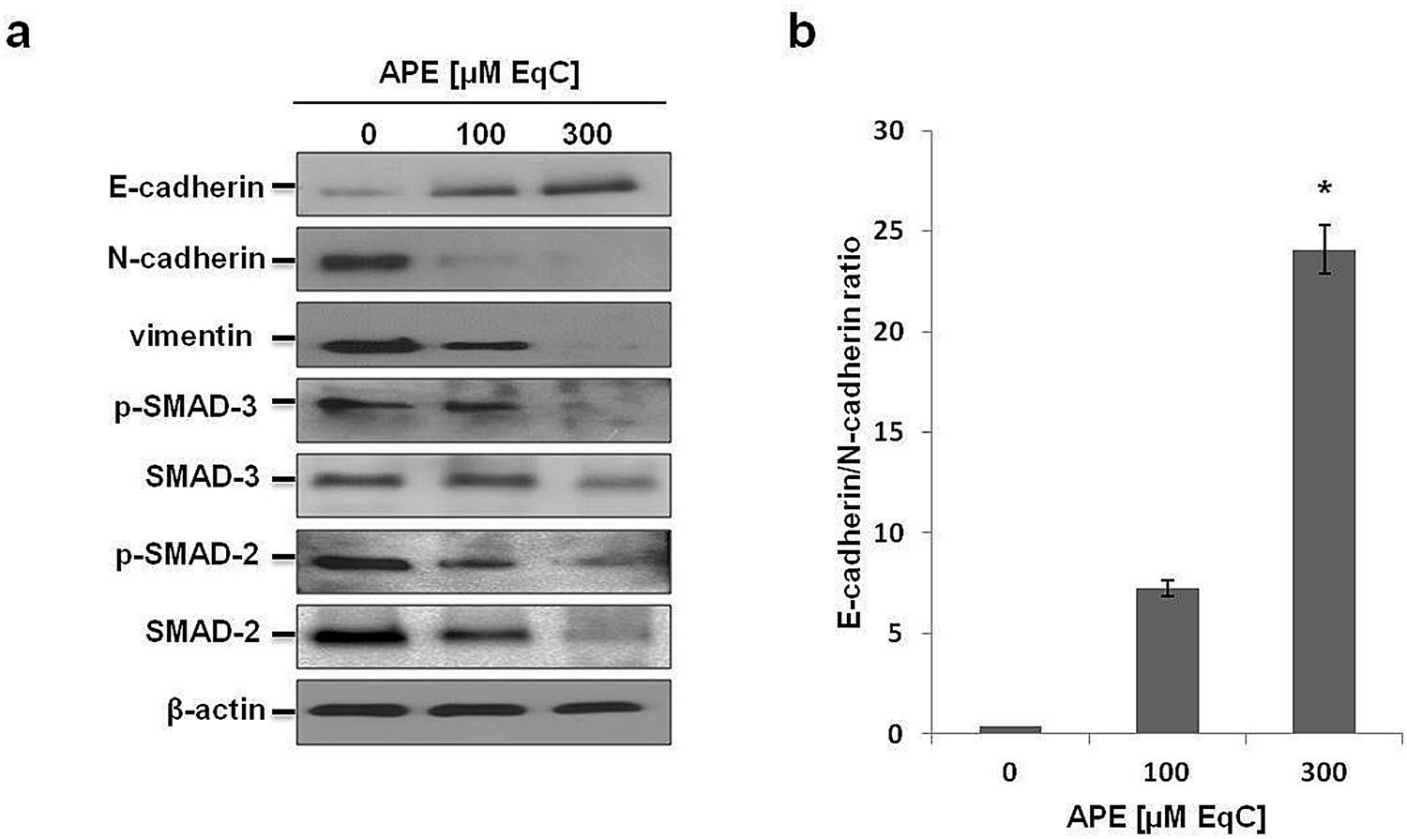
Effect of APE on the levels of EMT-related proteins in MDA-MB-231 cells. (**a**) The levels of EMT-associated protein markers in MDA-MB-231 cells treated with APE 100 and 300 μM EqC for 48 h were measured by Western blot. β-actin was used as a standard for the equal loading of protein in the lanes. The full-length blots are included in Supplementary Information (Fig. S4). (**b**) The graph shows the densitometric intensity of E-cadherin/N-cadherin ratio. The intensities of signals were expressed as arbitrary units (*P < 0.05 *versus* control cells).

EMT induces phenotypic changes enabling cells to lose epithelial characteristics and acquire motile mesenchymal traits^20^. MDA-MB-231 cells possess many features of a cell that has undergone EMT and do not express epithelial markers^21^, but, in contrast, are characterized by a high vimentin level^22^. To investigate whether the suppression of MDA-MB-231 cell migration upon APE treatment was associated with EMT inhibition and MET induction, the expression patterns of EMT-associated protein markers have been analyzed by Western blot (Fig. 2a). We firstly examined Small Mother Against Decapentaplegic (SMADs) protein status. SMADs are proteins that modulate the activity of TGF-β ligands and are critically important for regulating cell development, growth, and EMT^23^. Besides, MMP-2 and MMP-9, key enzymes involved in cell migration, are downstream target proteins regulated by TGF-β/SMAD signaling^24^. Our findings indicated that the levels of SMAD-2, SMAD-3, and their phosphorylated forms decreased in a dose-dependent way following APE treatment. Then, we analyzed vimentin, a type III intermediate filament and a major cytoskeletal component of motile mesenchymal cells, including metastatic tumor cells of epithelial origin^25^. Western blot analyses confirmed that MDA-MB-231 cells express high levels of vimentin which, after 48 h treatment with APE 300 μM EqC, became only slightly detectable suggesting APE-induced modifications of mesenchymal cell phenotype. Finally, we evaluated the levels of N-cadherin and E-cadherin, and we measured the E-cadherin/N-cadherin protein ratio. Our data exhibited a strong dose-dependent downregulation of N-cadherin and a noticeable up-regulation of E-cadherin resulting in a marked increase of E-cadherin/N-cadherin ratio that, at 300 μM EqC APE, increased about 25-fold compared to untreated cells (Fig. 2b). Overall, these results indicated that in MDA-MB-231 cells APE decreased the levels of the mesenchymal markers vimentin and N-cadherin, and modulated the TGF-β pathway via downregulation of SMAD-2/3 expression and phosphorylation causing the inhibition of EMT in turn responsible for the suppression of cell migration. Moreover, the significantly increased E-cadherin/N-cadherin protein ratio suggested that APE modulated EMT/MET-related markers strongly favoring MET.

### APE inhibited MDA-MB-231 cell migration through ROS/JNK signaling activation

**Figure 3:**
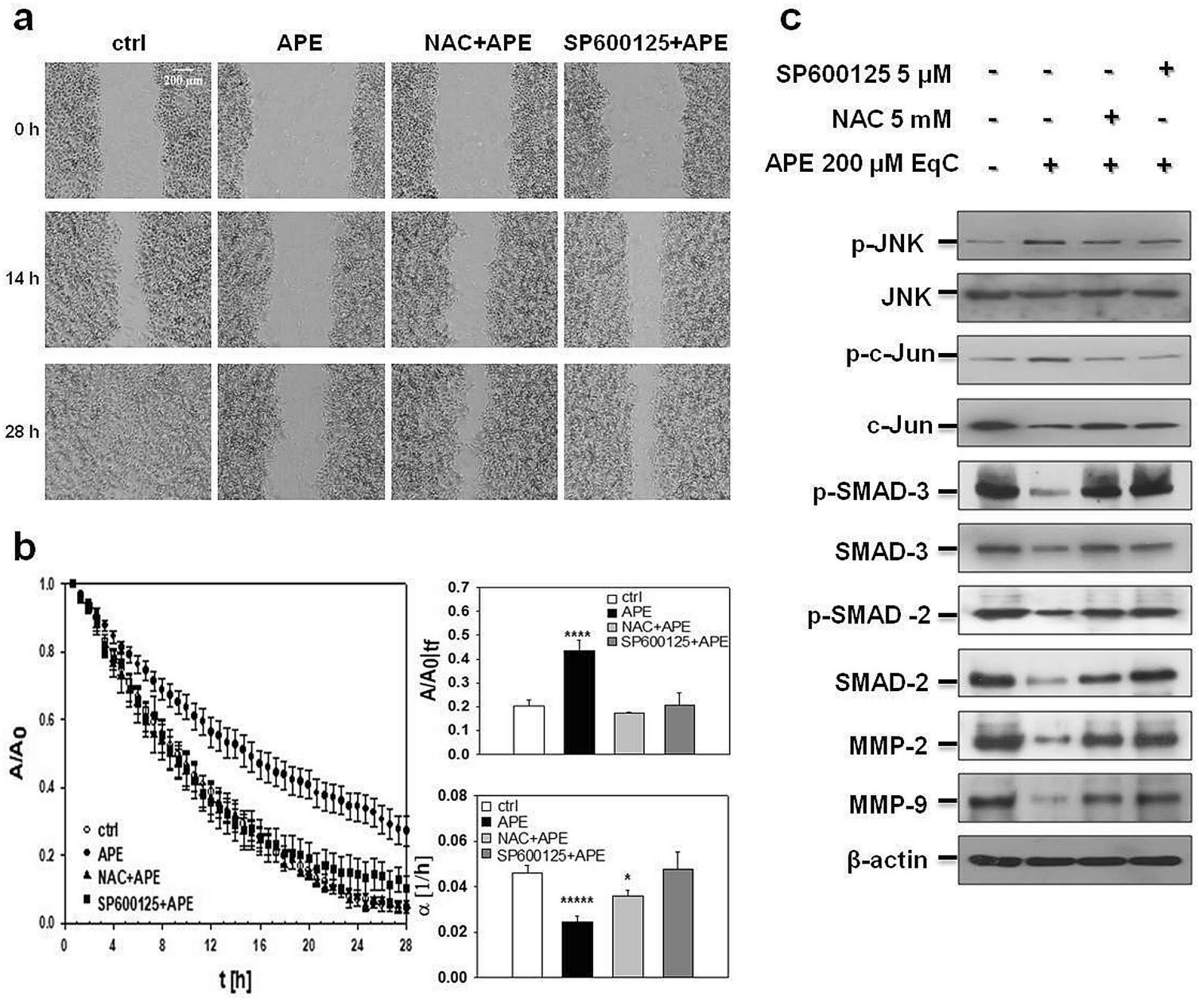
APE inhibited MDA-MB-231 cell migration through ROS/JNK signaling activation. (**a**) Representative phase-contrast microscopy images showing the wound closure process at three different times in MDA-MB-231 cells incubated or not (ctrl) with APE 200 μM EqC with or without 1 h pretreatment with 5 mM NAC and/or 5 μM SP600125. Representative time lapse videos (videos S9–S12) are included in Supplementary Information. (**b**) Evolution over time of the wound area A, normalized to the value at time 0 (A_0_), for MDA-MB-231 cells incubated or not (ctrl) with APE with or without 1 h pretreatment with NAC and/or SP600125. The linear range of each data series was fit in order to measure the wound closure velocity α. Upper bar diagram shows the A/A_0_ values for treated cells at the time t_f_, when the A/A_0_ value for control is 0.2. Lower bar diagram shows the values of α (h^-1^) for control and treated cells at time t_f_. The values reported in the histograms represent the mean from several independent fields of view. Standard error of the mean was calculated and one-tailed and heteroscedastic t-test were computed to verify the statistical significance of the differences with respect to the control samples (*P < 0.05, ****P < 5 × 10^–5^, *****P < 5 × 10^–6^). (**c**) Cells were treated with 200 μM EqC APE for 48 h with or without 1 h pretreatment with 5 mM NAC and/or 5 μM SP600125. Cell lysates were prepared and analyzed by Western blot for the expression of p-JNK, JNK, p–c-Jun, c-Jun, p-SMAD-3, SMAD-3, p-SMAD-2, SMAD-2, MMP-2, and MMP-9. All results were obtained from at least three independent experiments. The full-length blots are included in Supplementary Information (Figs. S5, S6).

To investigate whether APE-induced inhibition of MDA-MB-231 cell migration was dependent on ROS/JNK signaling activation, cells were pretreated 1 h with ROS scavenger N-acetyl-L-cysteine (NAC) and JNK inhibitor SP600125 before treatment with APE 200 μM EqC. Time-lapse analysis of the wound closure revealed that both molecules almost completely reverted the inhibitory effect of APE as indicated by the representative images from video sequences of the process (Fig. 3a and videos S9–S12). The evolution of the wound area overtime was reported in Fig. 3b. After pretreatment with NAC and SP600125, APE-treated cells filled the gap faster than without pretreatment indicating that ROS and JNK played a role in regulating cancer cell motility. Comparative analysis of ${\left.\frac{A}{{A}_{0}}\right|}_{{t}_{f}}$ (Fig. 3b, upper bar diagram) and wound healing velocity (α) values measured at time t_f_ (Fig. 3b lower bar diagram) in MDA-MB-231 cells incubated with APE 200 μM EqC with or without pretreatment with NAC and SP600125, respectively, showed that in pretreated cells both the wound size and the wound closure velocity returned to the values of the control cells confirming that the activation of ROS/JNK signaling represented the mechanism underlying APE-induced inhibition of TNBC cell migration. Notably, this result was supported by the evidence that NAC and SP600125 restored both the levels of relevant migration protein markers SMAD-2, p-SMAD-2, SMAD-3, p-SMAD-3, MMP-2, and MMP-9 (Fig. 3c) and the values of migration times of untreated cells (Fig. S2).

### APE induced MET in MDA-MB-231 cells by promoting the switch from N- to E-cadherin expression through ROS/JNK signaling activation

**Figure 4:**
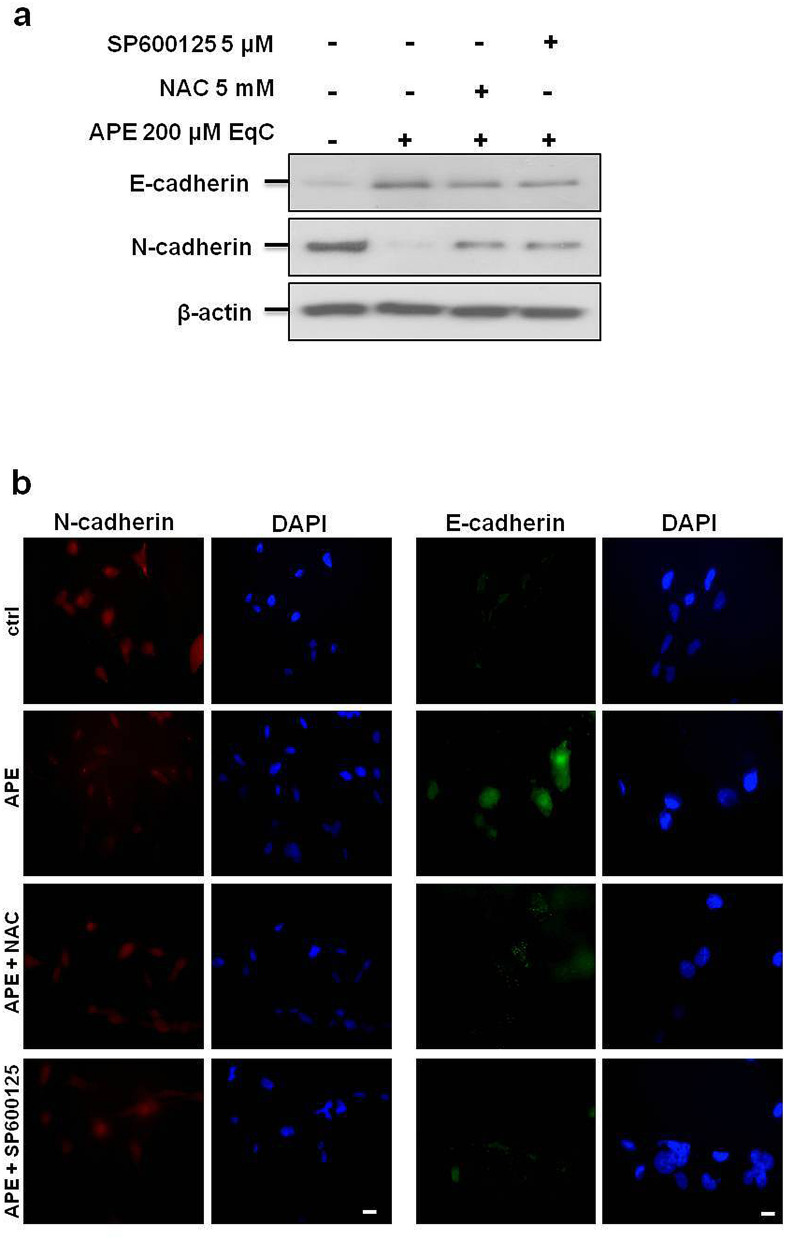
ROS/JNK signaling activation mediated APE-induced switch from N- to E-cadherin expression. (**a**) The levels of E-cadherin and N-cadherin in MDA-MB-231cells treated with APE 200 μM EqC for 48 h with or without 1 h pretreatment with 5 mM NAC and/or 5 μM SP600125 were measured by Western blot. β-actin was used as loading control. Results were obtained from at least three independent experiments. The full-length blots are included in Supplementary Information (Fig. S5). (**b**) Representative immunofluorescence images of N-cadherin and E-cadherin in MDA-MB-231 cells treated or not (ctrl) with 200 μM EqC APE for 24 h, with or without 1 h pretreatment with 5 mM NAC and/or 5 μM SP600125 are shown. Nuclei were stained with DAPI (blue), while anti N-cadherin (red) or E-cadherin (green) antibodies were used to detect the corresponding proteins. Images are representative of three independent experiments. Scale bar, 5 μm.

In tumor cells E- to N-cadherin switch is associated with increased migratory and invasive behavior and is believed of prognostic significance^26^. To assess whether APE-induced inhibition of cell migration was associated with E- to N-cadherin switch and to investigate the possible involvement of ROS/JNK signaling, MDA-MB-231 cells were incubated for 24 h with APE 200 μM EqC with or without 1 h pretreatment with NAC and SP600125. As shown in Fig. 4a, following APE treatment E-cadherin protein band that appeared barely noticeable in control cells, significantly increased while N-cadherin band became poorly detectable when compared with the large band observable in untreated cells. Confirming the Western blot results, immunofluorescence staining showed that the signal intensity of E-cadherin that was almost undetectable in untreated cells became evident in treated cells, while N-cadherin behaved in the opposite way resulting in N- to E-cadherin switch suggestive of APE-induced MET (Fig. 4b). Noteworthy, NAC and SP600125 brought the protein levels and the fluorescence intensities of E-cadherin and N-cadherin back to the control values indicating that ROS/JNK signaling activation represented the mechanism underlying APE-induced transition from N- to E-cadherin expression in MDA-MB-231 cells.

### APE induced MET in MDA-MB-231 cells by modifying vimentin expression and filamentous vimentin network through ROS/JNK signaling activation

**Figure 5:**
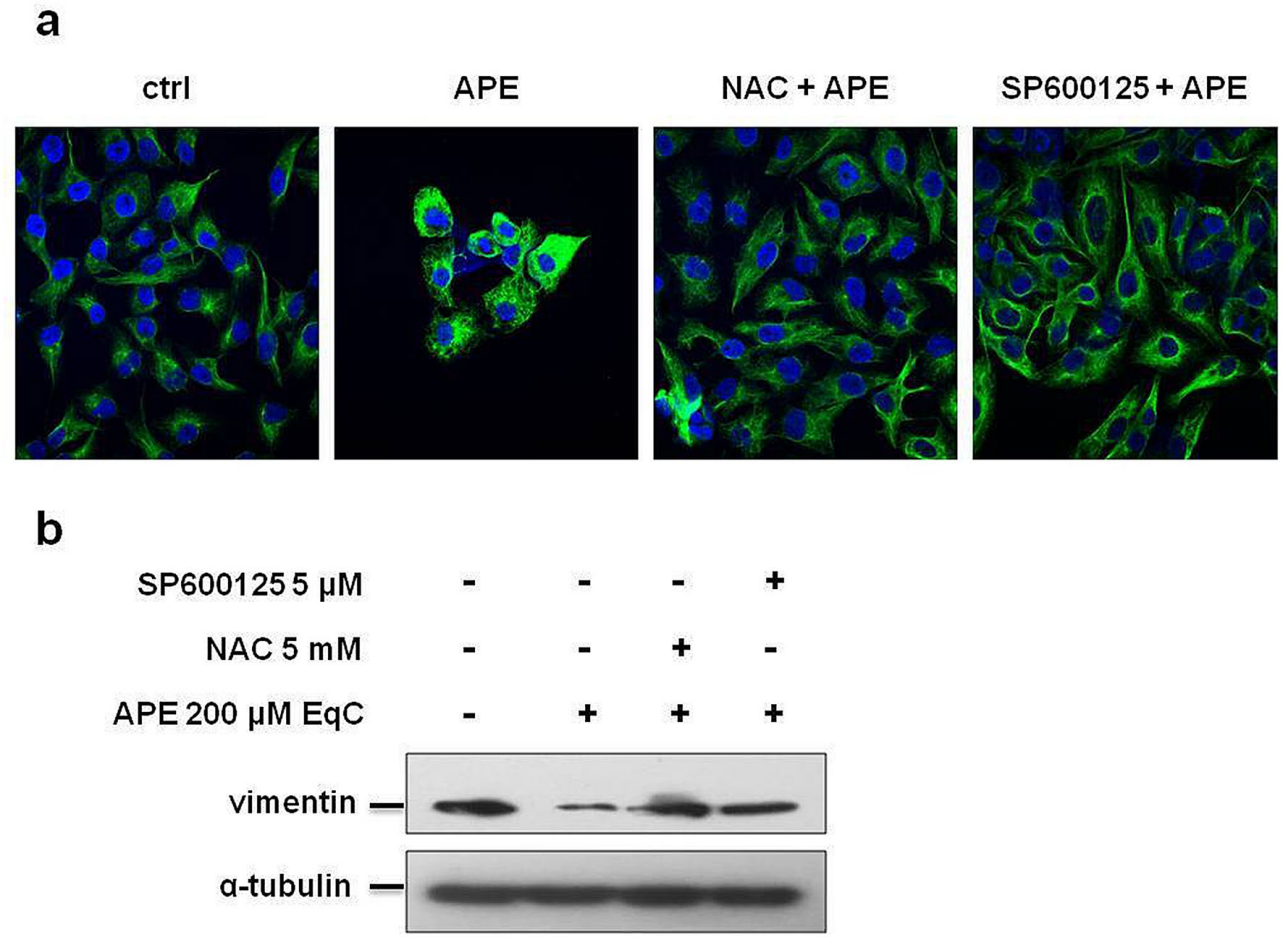
ROS/JNK signaling activation mediated APE-induced vimentin downregulation and vimentin network organization. (**a**) Representative confocal images of vimentin filament network in MDA-MB-231 cells treated or not (ctrl) with 200 μM EqC APE for 24 h, with or without 1 h pretreatment with 5 mM NAC and/or 5 μM SP600125. Nuclei were stained with DAPI (blue) while vimentin antibody (green) was used as cytoskeleton marker. (**b**) Western blot analysis of vimentin level in MDA-MB-231 cells treated with APE 200 μM EqC for 48 h with or without 1 h pretreatment with 5 mM NAC and/or 5 μM SP600125. The full-length blots are included in Supplementary Information (Fig. S6). All results were obtained from at least three independent experiments.

Vimentin is overexpressed in most epithelial cancers and its levels correlate with tumor migration, invasion, and poor prognosis. Drugs inhibiting or reversing EMT also affect vimentin network organization^25^. Vimentin plays an important role in the formation of lamellipodia and filopodia^27^, specialized actin-rich plasma membrane protrusions with proteolytic activity, that allow cancer cells to degrade ECM with directional movement enabling cell invasion^28^. To investigate whether APE may induce changes in the filamentous vimentin network and to probe the involvement of ROS/JNK axis in this process, we carried out a quantitative and qualitative assessment of vimentin by Western blot and immunofluorescence analysis. Confocal microscopy images (Fig. 5a) evidenced that untreated cells lacked cell–cell contacts and exhibited morphological features characteristic of mesenchymal cells such as membrane protrusions and elongated cell structure. On the other hand, after 24 h treatment with APE, vimentin filaments reorganized into perinuclear bundles, protrusions were no longer visible and cell–cell contacts became evident highlighting that APE triggered the transition of MDA-MB-231 cells from the mesenchymal to epithelial state (MET). Pretreatment with SP600125 and NAC restored the initial cell morphology demonstrating that JNK and ROS acted as important mediators of APE-induced morphological changes. Western blot analysis (Fig. 5b) revealed a significant decrease of vimentin levels in APE-treated cells that was reversed by pretreatment with SP600125 and NAC furnishing evidence that ROS and JNK modulated vimentin expression.

### APE induced MET in MDA-MB-231 cells by promoting actin cytoskeleton remodeling and changes of cell morphology through ROS/JNK signaling activation

**Figure 6:**
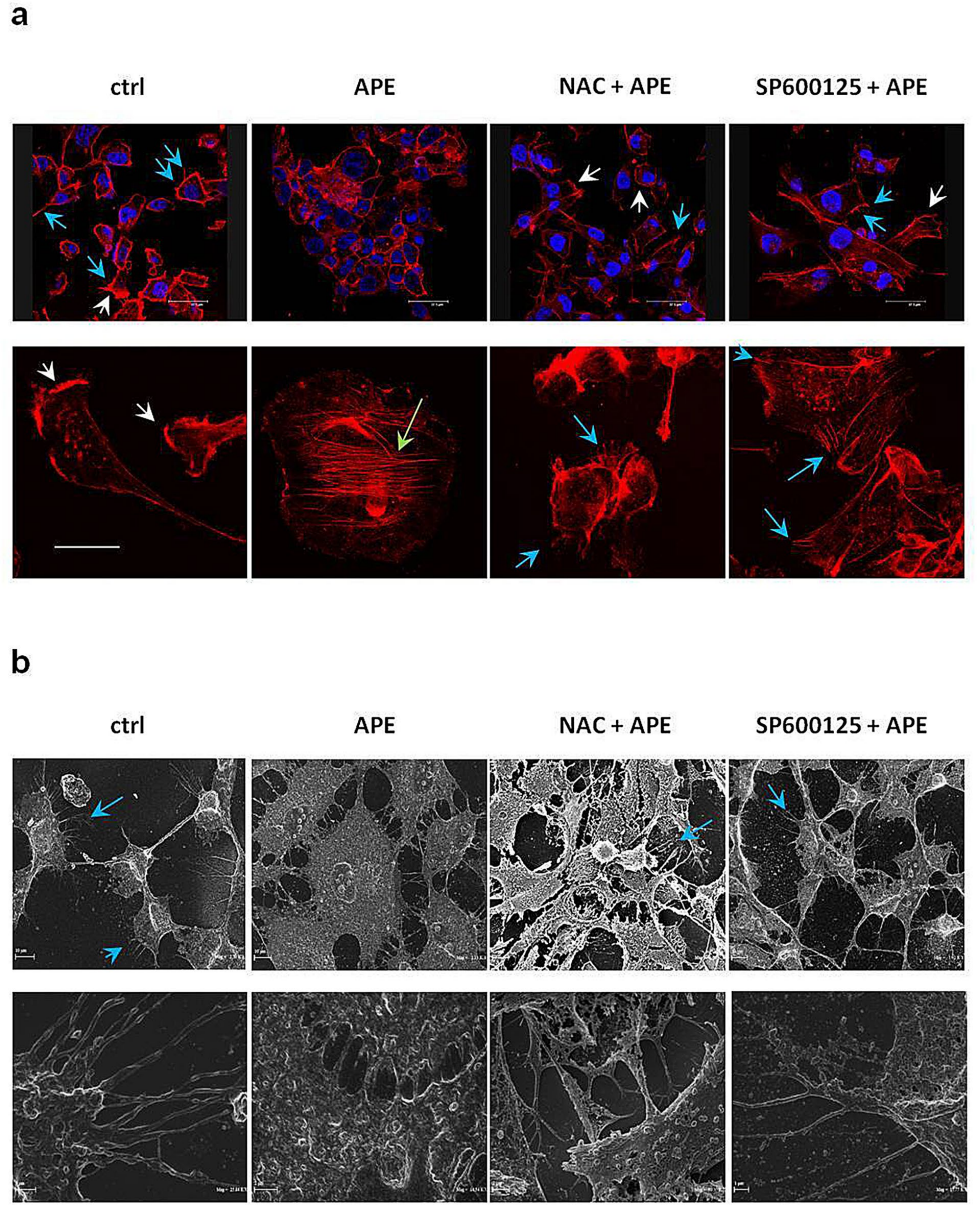
ROS/JNK signaling activation mediated APE-induced changes in podia formation, actin cytoskeleton remodeling, and cell morphology. (**a**) Representative confocal images of F-actin architecture in MDA-MB-231 cells treated or not (ctrl) with APE 200 µM EqC for 24 h with or without 1 h pretreatment with 5 mM NAC and/or 5 μM SP600125. Cells were cultured on fibronectin-coated coverslips and stained with Alexa Fluor 546 Phalloidin. Lamellipodia (white arrows), stress fibers (green arrows), and filopodia (blue arrows) were identified under a confocal microscope. Scale bar: top row, 37.5 μm; bottom row, 20 μm. (**b**) SEM images of MDA-MB-231 cells treated or not (ctrl) with APE 200 µM EqC for 24 h with or without 1 h pretreatment with 5 mM NAC and/or 5 μM SP600125. Control and pretreated cells showed typical mesenchymal characteristics such as elongated spindle-like shape with cytoplasmic protrusions like filopodia (blue arrow) and few globular rounded cells. APE treated cells showed a wide cytoplasm, tight cell–cell contacts, and an increase of flattened very large cuboid cells which appeared more grouped, resembling epithelial-like sheets. Scale bar: top row, 10 μm; bottom row, 1 and 2 μm.

The modification of cell morphology occurring during EMT is associated with progressive and dynamic remodeling of the intracellular architecture of filamentous actin (F-actin) including changes in bundling and contractility of stress fibers and emission of membrane protrusions that allow cancer cells to migrate to distant sites where they may establish metastases^29,30^. To obtain more direct evidence of APE-induced MET in MDA-MB-231 cells, we analyzed actin cytoskeleton organization and podia-parameters by confocal microscopy after labeling of F-actin with phalloidin red and we performed high-resolution cell imaging by scanning electron microscopy (SEM) to better highlight changes of cell morphology occurring after APE treatment. As indicated by fluorescent actin images (Fig. 6a), untreated cells showed an elongated and spindle-shaped morphology with large protruding lamellipodia followed by the main cell bodies. In contrast, after treatment with APE for 24 h, cells became cuboidal-shaped and lamellipodia were no longer detectable. Interestingly, the marked changes in cell morphology induced by APE were accompanied by the presence of stress fibers. This finding is suggestive of cells undergoing MET based on reports showing that stress fibers are characteristic of strong cell adhesion^31^ and that stress fiber organization promotes cell stiffening and proliferation of pre-invasive breast cancer cells^32^. Notably, pretreatment with NAC and SP600125 highlighted the appearance of filopodia, actin-containing spikes with sensory or exploratory functions that have roles in migration and contribute to invasion and metastasis of cancer cells^33^. Morphological SEM analysis reported in Fig. 6b, confirmed the mesenchymal pattern of MDA-MB-231 cells, with an elongated shape body and many filopodia. Contrariwise, SEM images of APE-treated cells showed a cell population mostly including very large cuboidal cells, with a wide cytoplasm and evident cell–cell contacts forming sheet-like monolayers resembling epithelial cells. Confirming the results of confocal microscopy, SEM analysis highlighted that most of NAC and SP600125 pretreated cells regained a spindle-like morphology and the presence of filopodia while just a sparingly number exhibited a globular rounded shape. Altogether these results provided direct and convincing evidence that APE triggered MET in MDA-MB-231 cells by inducing morphological changes resulting in a cell phenotype with decreased migratory behavior. The ability of NAC and SP600125 to restore the original mesenchymal cell phenotype represented the final and conclusive evidence that the induction of MET by APE is dependent on ROS/JNK signaling activation.

## Discussion

Aberrant activation of EMT and stemness features have been reported to be responsible for TNBC cell invasion, metastasis, and resistance to treatments^34^. On this basis, the identification of bioactive molecules able to suppress tumor cell dissemination without causing significant toxic side effects represents an important clinical challenge. New advances in anticancer drug discovery using natural products highlighted APE as a multi-faceted agent that selectively kills TNBC cells through generation of toxic levels of ROS, sustained JNK/c-Jun signaling activation, and inhibition of pathways involved in cell growth and survival^15^. The novel finding of the current study, obtained by recording the real-time movement of live cells in wound healing assay and by monitoring migration- and EMT-related molecular and morphological parameters, indicated that APE inhibited TNBC cell migration and induced MET in MDA-MB-231 cells driving their mesenchymal-like phenotype toward an epithelial morphology with reduced migratory features and that the activation of ROS/JNK axis represented the main underlying molecular mechanism.

We found that APE, under experimental conditions where only poor effect on cell viability was observed, caused a dose-dependent decrease in the migration rate of MDA-MB-231 and MDA-MB-468 cells indicative of its ability to lower the aggressiveness of TNBC cells and to potentially reduce their metastatic power.

Cancer cells undergoing metastasis express MMPs, a family of zinc-dependent endopeptidases able to degrade almost all components of ECM and basement membrane. Considerable experimental and clinical evidence documents that MMPs are involved in several steps of cancer development beside cell invasion and metastasis thus representing ideal pharmacological targets in tumor therapy^17^. Overexpression of MMPs has been associated with unfavorable outcomes in several malignant tumors, including breast^35,36^. Recent literature reported that naturally-derived plant polyphenols can block migration and invasion of breast cancer cells through a series of molecular mechanisms, including the downregulation of MMPs^37^. Accordingly, we showed that APE-induced inhibition of TNBC cell migration was associated with a dose-dependent decrease of MMP-2 and MMP-9 and that pretreatment of cells with NAC and SP600125 resulted in the reversion of both events indicating the involvement of ROS/JNK signaling in these processes.

EMT is an evolutionary conserved program where, under physiological or pathological conditions, immotile epithelial cells are transcriptionally reprogrammed to acquire a mesenchymal phenotype allowing them to move in the ECM. In cancer cells, EMT is associated with increased aggressiveness and invasive and metastatic potential and is also involved in the generation of tumor stem-like cells that play a major role in chemoresistance^38^. During EMT the genes encoding epithelial junction proteins, such as E-cadherin, become downregulated leading to loss of apical-basal polarity and disassembling of adherent junctions involved in the extensive cell adhesions to neighboring cells and basement membrane. On the contrary, genes associated with the mesenchymal phenotype such as vimentin and N-cadherin are upregulated^39^. An interesting feature of the EMT is its potential reversibility at any time by simply changing the expression of key molecular components. Indeed, disseminated cancer cells, under influences from their microenvironment, may undergo MET facilitating their integration at the secondary tumor site and enabling metastatic colonization^40^.

MDA-MB-231 cells belong to the mesenchymal subtype of TNBC and are enriched in genes involved in EMT such as Wnt and TGF-β pathways^41^. Owing to their mesenchymal phenotype and aggressive and metastatic features, MDA-MB-231 cells have been utilized as experimental model to investigate MET induction in TNBC. We provided here evidence that APE was able to promote MET in MDA-MB-231 cells, as demonstrated by the observed APE-induced changes in cellular phenotype and key EMT-related molecular markers.

First, cadherins are homophilic adhesion molecules acting as crucial regulators of tumor development^39^. N-cadherin is highly expressed in mesenchymal cells and neural tissues^42^. N-cadherin upregulation is considered an important marker of ongoing EMT and has been shown to promote motility and invasion of tumor cells^43^. On the other hand, E-cadherin plays an important role in maintaining phenotype and polarization of the epithelial cell layers. In tumor cells, the loss of E-cadherin-mediated cell adhesions correlates with the loss of epithelial morphology and with increased invasiveness and metastatic dissemination^42,44^. The observation that E-cadherin is re-expressed in metastatic tumor cells^45^ and that the ectopic expression of E-cadherin in MDA-MB-231 cells was associated with morphological and functional reversion of the mesenchymal phenotype^46^ strongly indicates that E-cadherin is a hallmark of MET and an inducer of this process. On this basis, identifying new compounds able to restore an epithelial-like phenotype by E-cadherin upregulation could represent a suitable approach for treating invasive TNBC. Notably, we found that APE induced N- to E-cadherin switch in MDA-MB-231 cells evidenced by increased E-cadherin/N-cadherin protein ratio and by the shift of immunofluorescence signal from N-cadherin to E-cadherin in APE-treated cells. Based on the view that the E- to N-cadherin switch is thought a hallmark of EMT^26^ it is conceivable to consider the observed reverse N- to E-cadherin switch as indicative of APE-induced MET in MDA-MB-231 cells.

Second, tumor cells undergoing EMT significantly modify the composition of cytoskeletal intermediate filaments with the repression of keratin and expression of vimentin that is believed to be responsible for the adoption of a mesenchymal shape and increased motility and is regarded as the main and conventional canonical marker of EMT^22^. In line with this evidence, it has been shown that using small interfering RNA or a dominant-negative vimentin probe attenuated the migration of many tumor cells and resulted in re-expression of epithelial keratins, allowing cancer cells to partially restore their epithelial phenotype^47,48^. The crucial role played by vimentin in regulating the mechanical, migratory, and invasive properties of cancer cells has been recently highlighted by studies showing that vimentin-lacking MDA-MB-231 cells are softer, more deformable, and less contractile and lose directional persistence of migration^22^. Interestingly, we demonstrated that APE downregulated the levels of vimentin in MDA-MB-231 cells and caused a significant rearrangement of the vimentin network.

Reorganization of the actin cytoskeleton and remodeling of actin-rich membrane projections which contribute to cell directional motility represent important steps for cell transition from epithelial to mesenchymal state. Noteworthy, we found that APE caused evident modifications in the overall architecture of filamentous actin dramatically modifying the shape and motile features of MDA-MB-231 cells that underwent a visible transition from a mesenchymal to epithelial phenotype with decreased migratory and invasive behavior. Notably, the increased levels of E-cadherin, the decreased levels of N-cadherin and vimentin, and the structural and morphological changes observed following APE treatment were reversed by NAC and/or SP600125 indicating that the activation of ROS/JNK signaling cascade mainly contributed to APE-induced MET in MDA-MB-231 cells.

Third, TGF-β is constitutively expressed in metastasizing breast carcinoma and is widely recognized as the most potent inducer of EMT process and a key mediator for metastasis into other tissues during breast cancer progression^5^. On this basis, the development of drugs targeting TGF-β signaling may be an effective strategy to suppress breast cancer cell migration and metastasis^49^. SMAD signaling is crucially involved in TGF-β-induced EMT that is in turn interconnected with MET in a time-dependent and tissue context-dependent manner^50^. Interestingly, we found that APE significantly decreased the protein levels of SMAD-2/3 and their phosphorylated forms supporting the hypothesis that TGF-β/SMAD pathway could represent one of the targets selected by APE to trigger MET in MDA-MB-231 cells. Notably, the decrease of SMAD-2/3 and p-SMAD-2/3 levels was reversed by NAC and/or SP600125 providing evidence that ROS/JNK signaling mediated APE-induced MET in MDA-MB-231 cells by modulating TGF-β/SMAD signaling.

Many signaling pathways and oncoproteins, including AKT, p21, NF-kB, c-myc, and β-catenin have established roles in migration and EMT progression of TNBC cells; (1) activation of NF-kB signaling stimulates an aggressive phenotype of breast cancer cells through the transcriptional activation of EMT regulatory genes. Blocking NF-κB/p65 activity in MDA-MB-231 cells promotes upregulation of E-cadherin leading to decreased cell migration and invasion^51^; (2) p21 acts as a transcriptional co-regulator of SMAD in mediating TGFβ-induced breast cancer cell migration. Silencing of p21 gene caused the inhibition of tumor invasion in a mammary fat pad xenograft mouse model and in various breast cancer cell lines^52^; (3) activated AKT signaling represents one of the most commonly observed dysregulations in breast cancer and has established roles in migration and metastasis of TNBC cells making inhibition of AKT an attractive therapeutic target^53,54^. The evidence that in breast carcinoma the activity of AKT was repressed during MET suggests a relationship between AKT inhibition and MET^55^; (4) c-myc is an important transcription factor overexpressed in aggressive TNBC where its deregulation contributes to disease progression, metastatic potential, and therapeutic resistance^56,57^. It has been reported that c-myc expression in human mammary epithelial cells repressed E-cadherin expression, increased vimentin expression and induced EMT associated to dramatic changes in cell morphology^58^; (5) *β*-catenin is a multifunctional protein that serves as an essential component of adherent junctions and represents the most important mediator of the canonical Wnt pathway. Aberrant activation of Wnt pathway results in the release of *β-*catenin from the membrane and its accumulation in the nucleus where it promotes the transcription of many Wnt target genes involved in several tumor-associated properties, including EMT and cell migration^59^. Inhibition or silencing of β-catenin markedly inhibits the expression of EMT-related transcriptional factors resulting in the reversal of EMT and alleviation of breast cancer metastasis^60^. Based on all these literature data, reverting EMT process by inhibiting its signaling is considered an appealing strategy in cancer treatment. It has to be pointed out that natural polyphenols have been reported to revert or suppress cancer invasion and metastasis through inhibition of EMT-related signaling pathways in various cancer cells^9^. Moreover, many compounds that reverse EMT in breast cancer have been used in preclinical studies^61^. Interestingly, we have previously demonstrated that in MDA-MB-231 cells APE downregulated AKT, p21, NF-kB, c-myc, and β-catenin (Fig. S7) and that pretreatment with NAC and SP600125 restored β-catenin and p21 level while SP600125 reverted NF-kB downregulation^15^ (Fig. S8). In the whole, these findings supported our idea that APE could be considered a promising novel natural agent for the selective targeting of EMT in TNBC cells and further confirmed that ROS and JNK acted as mediators of APE-induced reversion of EMT.

**Figure 7:**
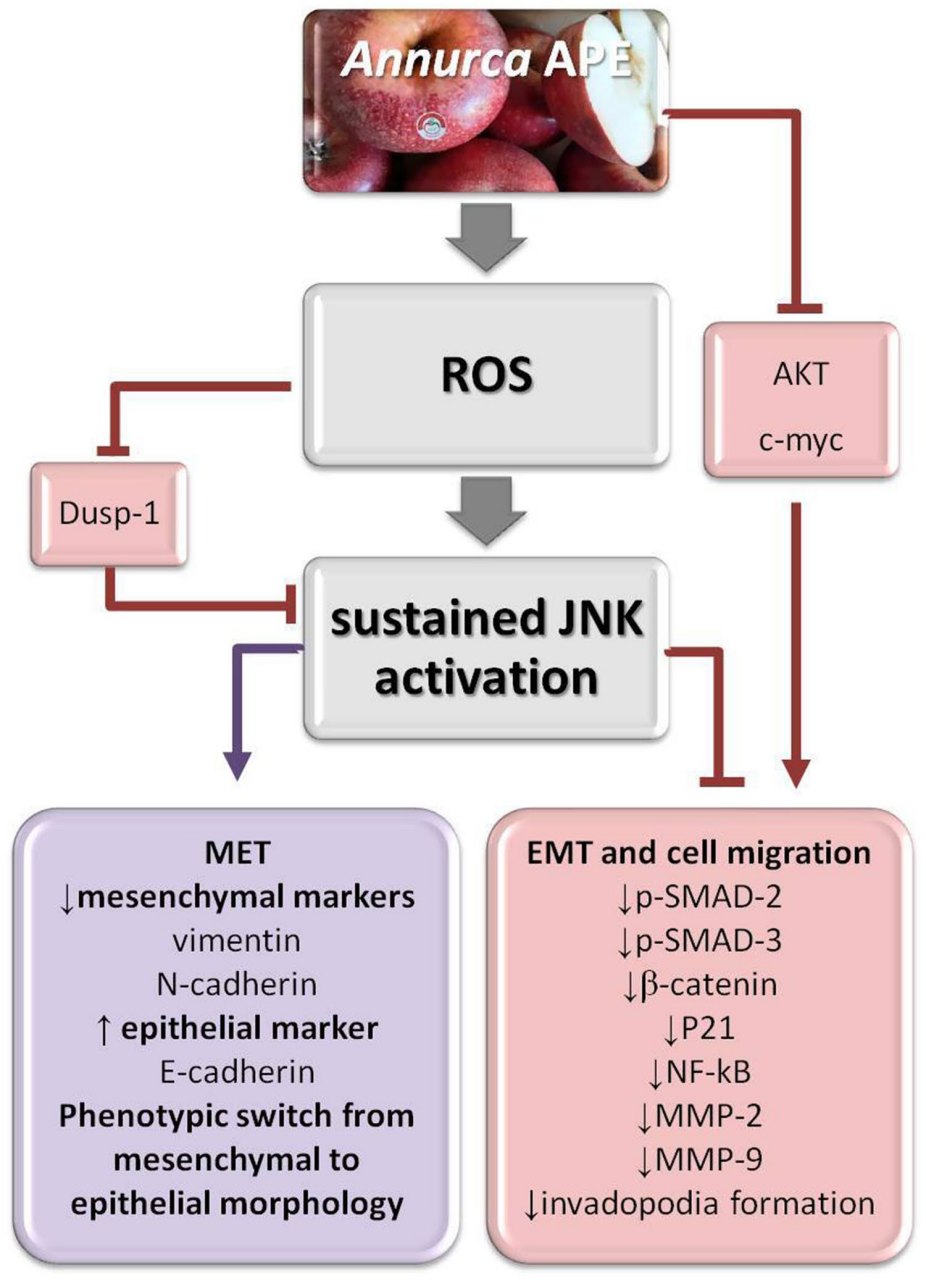
Schematic representation of the molecular events involved in *Annurca* APE-mediated effects on cell migration and mesenchymal-to-epithelial transition in MDA-MB-231 cells. The photo of *Annurca* apple was taken by the authors. Cropped and full-length blots for AKT, p-AKT, c-myc, Dusp-1, β-catenin, p-21, and NF-kB are included in Supplementary Information (Figs. S7, S8).

**Figure 8:**
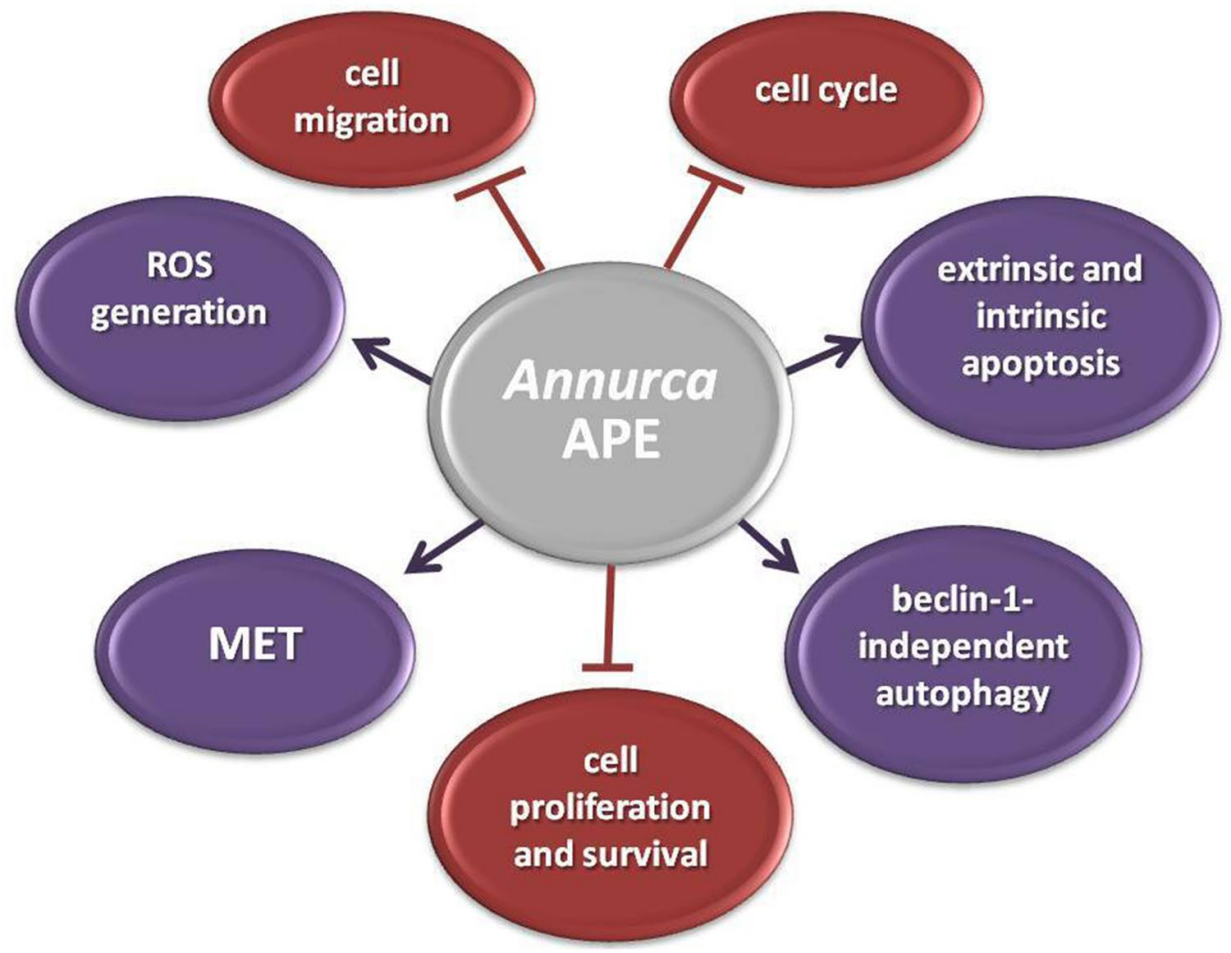
Multi-targeted anticancer effects of *Annurca* APE in MDA-MB-231 cells.

In conclusion, our study for the first time elucidated the effects exerted by APE on MET and cell migration in MDA-MB-231 cells. As schematically represented in Fig. 7 increased ROS generation represents an early and crucial event. Next, JNK activation, sustained in magnitude and duration by ROS-triggered inhibition of dual-specificity phosphatase-1 (Dusp-1)^62^ (Fig. S8) and further potentiated by positive feedback loops^15^, plays a key role downstream of ROS in mediating both inhibition of cell migration and MET induction. The ability of APE to decrease the invasive potential of MDA-MB-231 cells by triggering a shift from a mesenchymal to epithelial phenotype via reversal of the EMT state to the MET state should prevent the metastatic spread of these aggressive TNBC cells. It has to be noted that MET induction is not without consequences, because cells that undergo MET might support the process of metastatic colonization at distant sites^63^. In this respect, APE can be considered a very promising agent being able to promote MET in MDA-MB-231 cells and, at the same time, to lower their migratory and proliferative features. As highlighted by the picture in Fig. 8, the ability to promote MET, to suppress cell migration, to arrest cell cycle^15^, to selectively kill tumor cells by inducing apoptotic and autophagic death^15^, and to inhibit growth and survival pathways while exerting protective antioxidant effects in normal cells^15^, render APE an encouraging candidate for TNBC treatment.

## Methods

### Chemicals and antibodies

NAC, 3-(4,5-dimethylthiazol-2-yl)-2,5-diphenyltetrazolium bromide (MTT), Folin-Ciocalteu reagent, and 4′,6-diamidino-2-phenylindole (DAPI) nucleic acid stain were purchased from Sigma-Aldrich (St. Louis, MO). SP600125 JNK inhibitor was obtained from Selleck Chemicals (Munich, Germany). Monoclonal antibodies to SMAD-2, SMAD-3, p-SMAD-3, MMP-9, N-cadherin, vimentin, p–c-Jun, c-Jun, β-actin, α-tubulin, and polyclonal antibodies to MMP-2 and p-SMAD-2 were purchased from Cell Signaling Technology (Danvers, MA). Monoclonal antibodies to p-JNK and JNK were from Santa Cruz Biotechnology (Dallas, USA). The monoclonal antibody to E-cadherin was acquired from BD Biosciences (San Josè, USA). Horseradish peroxidase (HRP)-conjugated goat anti-mouse and HRP-conjugated goat anti-rabbit secondary antibodies were obtained from ImmunoReagents Inc. (Raleigh, NC). The secondary antibody Alexa Fluor 488 was from Invitrogen (Carlsbad, CA). Fluorescein isothiocyanate (FITC)-conjugated AffiniPure rabbit anti-mouse IgG and Texas Red dye-conjugated AffiniPure goat anti-rabbit IgG were provided from Jackson Immuno Research Laboratories (West Grove, PA). Alexa Fluor 546 Phalloidin was from ThermoFisher Scientific (Waltham, MA). All buffers and solutions were prepared with ultra-high quality water. All reagents were of the purest commercial grade.

### Apple samples and polyphenolic content

*Annurca* (*Malus pumila* Miller cv. *Annurca*) apple fruits are picked in October and, before the complete maturity, put in the “melai”, special boxes for the reddening on the ground. APE extraction from *Annurca* apple fruits (each weighing approximately 100 g) was carried out as previously reported^64^. The total polyphenolic content of apple extract was assessed by Folin-Ciocalteu phenol reagent as described by Singleton *et al.*^65^. Catechin was used as a reference standard and its value, expressed as milligrams of catechin equivalents (EqC)/100 g of flesh fresh weight, resulted in approximately 125.2 ± 7.1 mg of catechin per 100 g of sample. The determination of the polyphenolic profile of *Annurca* APE performed by HPLC analysis identified ( +)-catechin, (−)-epicatechin, chlorogenic acid, quercetin, and quercetin glycosides as the main *Annurca* apple *o*-diphenols confirming the results already described in the literature^14^.

### Cell culture

MDA-MB-231 and MDA-MB-468 cell lines were obtained from the American Type Culture Collection (ATCC, Manassas, VA). Cells were cultured in Dulbecco’s Modified Eagle Medium (DMEM) supplemented with 10% fetal bovine serum (FBS), antibiotics (100 U/ml penicillin and 100 μg/ml streptomycin) and 1% L-glutamine, and maintained in a humidified atmosphere containing 5% CO_2_ in the air.

### Cell viability

The effect of APE on cell viability was determined by the colorimetric MTT assay according to the manufacturer's instruction. Cell viability was measured as previously reported^14^. Briefly, MDA-MB-231 and MDA-MB-468 cells were seeded in serum-containing media in 96-well plates at the density of 4 × 10^3^ cells/well and treated with increasing APE concentrations. After 24 and 48 h, the incubation medium was removed and the MTT solution was added to a final concentration of 0.5 mg/ml. Cell viability was normalized as a percentage of control. All MTT experiments were performed in quadruplicate.

### Wound-scratch assay

MDA-MB-231 and MDA-MB-468 cells were seeded in serum-containing media in 12-well plates at a density of 4.5 × 10^5^ cells/well and incubated for 24 h until confluence. Cell monolayers were manually scratched with a sterile pipette tip to form a straight wound, washed twice with medium to remove cell debris, and incubated with APE 100, 200, and 300 μM EqC with and without 1 h pretreatment with 5 μM SP600125 and 5 mM NAC. Cell motility was assessed by time-lapse video microscopy.

### Time-lapse video microscopy

Wound closure was tracked by automated time-lapse video microscopy^68,69^ based on an inverted microscope (Zeiss Axiovert 200, Carl Zeiss, Jena Germany) enclosed in a homemade incubator that allows keeping the sample at a constant temperature (37 ± 0.1 °C), 5% CO_2_ and 100% humidified atmosphere. Several independent fields of view in each cell dish were acquired by a high-resolution high-sensitivity monochromatic CCD video camera (Hamamatsu Orca AG, Japan) using a long working distance 5 × objective in phase-contrast (CP Achromat Ph1), at regular intervals (20 min). The workstation was also equipped with motorized stage and focus control (Marzhauser) for automated positioning that allows to iteratively image specific regions along the wound and was controlled by homemade control software in Labview. The overall experiment duration was about 48 h but the significant results have been obtained within 28 h.

### Image and wound healing data analysis

The dynamic of wound healing process was quantified by measuring the area of the cell-free region for each time step (A), normalized to its value at time 0 (A_0_). The cell-free area was measured by identifying the wound edge as the boundary of the cell nude area through a homemade automated image analysis software based on the analysis of image variance. The comparison of wound healing kinetics for the control and treated samples was carried out by plotting A/A_0_ as a function of time. After an initial lag time (t_l_), the obtained profiles, all starting from A/A_0_ = 1, exhibited a linear trend, in agreement with the prediction of the Fisher-Kolmogoroff model^68^, suggesting that the wound healing process occurs at a constant velocity. The slope of the linear range of the A/A_0_ vs. t curve can be considered a measure of the wound closure velocity α (h^−1^)^69^. In Supplementary Information a typical experimental curve where A/A_0_ is plotted as a function of time for a control and a treated sample is described and graphically analyzed (Fig. S1). An additional and more simple method to compare the wound closure dynamics among different samples was the analysis of their ${\left.\frac{A}{{A}_{0}}\right|}_{{t}_{f}}$ corresponding to the the value of the wound closure A/A_0_ for each treatment at the time t_f_, defined as the time when the control reaches an 80% closure (A/A_0_ = 0.2). The contribution of cell motility and proliferation to wound healing was evaluated by calculating the Thiele modulus as described in Supplementary Information.

### Immunofluorescence staining

Cells were processed for immunofluorescence analysis as reported previously^15^. Briefly, MDA-MB-231 cells were cultured in 24-well plates containing microscope glass (12 mm) (Thermo Scientific) at 1.8 × 10^4^ cells/well and treated for 24 h with APE 200 μM EqC with and without 1 h pretreatment with 5 μM SP600125 and/or 5 mM NAC. Cells were then incubated for 1 h at 37 °C with a primary antibody to vimentin (1:1000) followed by incubation with secondary antibody conjugated to Alexa Fluor 488 (1:1000) or with primary antibody to E-cadherin and N-cadherin (1:100) followed by incubation with (1:200) FITC-conjugated AffiniPure rabbit anti-mouse and Texas red-conjugated anti-rabbit antibody, respectively. Nuclei were counterstained with DAPI. Vimentin images were performed using Zeiss LSM 700 confocal microscope equipped with a plan apochromat X63 (NA 1.4) oil immersion objective while for E-cadherin and N-cadherin imaging, the coverslips were inverted, mounted in Moviol (Calbiochem, CA) on glass slides, and analyzed using an Axiophot fluorescent microscope (Zeiss). Significant fields were captured and processed using a KS300 system (Zeiss).

### Polymerized F-actin visualization

MDA-MB-231 cells were cultured in *μ*-slide 8 well (IBIDI Systems, Munich, Germany) and treated with APE 200 µM EqC with or without pretreatment with 5 mM NAC and/or 5 μM SP600125. To visualize the polymerized F-actin, cells were fixed with 3.7% methanol-free formaldehyde for 20 min and permeabilized with 0.1% Triton X-100 in PBS for 10 min at room temperature. Then, the supernatant was removed and, after three times washing with PBS, the cells were incubated for 40 min with 0.1 μg/ml Alexa Fluor 546 Phalloidin. For nuclear staining the cells were incubated with 2.5 µg/ml DAPI for 7 min at room temperature in the dark. Microscopy images were performed using Leica Confocal Microscope (Laser Scanning TCS SP2) equipped with Kr/Ar and He/Ne lasers.

### Scanning electron microscopy

MDA-MB-231 cells were cultured in 24-well plates containing microscope glass (12 mm) (Thermo Scientific) at 1.8 × 10^4^ cells/well and treated for 24 h with APE 200 μM EqC with and without 1 h pretreatment with 5 μM SP600125 and/or 5 mM NAC. Samples were fixed with 3.7% methanol-free formaldehyde for 20 min and washed with PBS. Then, after dehydrating through graded ethanol concentrations, samples were critical point-dried in CO_2_ (EMITECH K850), platinum-palladium-coated by sputtering (Denton Vacuum DESKV), and observed with Supra 40 FESEM (Zeiss).

### Preparation of cell lysates and Western blot analysis

MDA-MB-231 and MDA-MB-468 cells were cultured in 10-cm culture dishes and treated for 48 h with different concentrations of APE with or without pretreatment with 5 mM NAC and/or 5 μM SP600125 for 1 h. Cells were processed for Western blot analysis as reported previously^15^. Cell lysates containing equal amounts of total proteins were separated by SDS-PAGE, electrotransferred onto nitrocellulose membranes by Trans blot turbo (Bio-Rad Laboratories), and subjected to standard immunoblotting. The blots were incubated with specific primary antibodies, washed, and incubated with HRP-conjugated secondary antibodies. Immunoblots were developed by using enhanced chemiluminescence detection reagents ECL (Westar, Cyanagen, Italy), exposed to X-ray film and scanned by ImageJ software (National Institutes of Health, Bethesda, MD, USA).

### Statistical analysis

All experiments were performed at least three times with replicate samples. Data are expressed as mean ± standard deviation (SD). Comparisons between treated samples and control were performed using analysis of variance (ANOVA) plus Bonferroni's t-test. Results were considered statistically significant at a *P* value < 0.05. Wound healing experiments were performed in triplicate. For each condition at least 12 independent fields of view were measured, obtaining A/A_0_ vs. t curves that where independently fit to calculate kinetic parameters. The significance of the differences between treated and untreated samples was determined by one-tailed and heteroscedastic t-test. Indicated *P* values are considered statistically significant.

## Supplementary information

Supplementary Information.

Supplementary Video 1.

Supplementary Video 2.

Supplementary Video 3.

Supplementary Video 4.

Supplementary Video 5.

Supplementary Video 6.

Supplementary Video 7.

Supplementary Video 8.

Supplementary Video 9.

Supplementary Video 10.

Supplementary Video 11.

Supplementary Video 12.
