# Supplementary material for: Annurca apple polyphenol extract promotes mesenchymal-to-epithelial transition and inhibits migration in triple-negative breast cancer cells through ROS/JNK signaling
Source: Sci Rep. 2020 Sep 28;10:15921. doi: 10.1038/s41598-020-73092-2 (PMC7522716; doi:10.1038/s41598-020-73092-2)
Supplement: Supplementary file 1 — Supplementary Information. [file 41598_2020_73092_MOESM1_ESM.pdf]

Supplementary Information

Annurca apple polyphenol extract promotes mesenchymal-to-epithelial transition and inhibits migration in triple-negative breast cancer cells through ROS/JNK signaling

Daniela Cristina Vuoso[1], Stefania D'Angelo[2], Rosalia Ferraro[3], Sergio Caserta[3], Stefano Guido[3], Marcella Cammarota[4], Marina Porcelli[1], and Giovanna Cacciapuoti[1*]

[1]Department of Precision Medicine, University of Campania "Luigi Vanvitelli", via Luigi De Crecchio 7, 80138 Naples, Italy. [2]Department of Motor Sciences and Wellness, "Parthenope" University, via Medina 40, 80133, Naples, Italy. [3]Department of Chemical, Materials and Industrial Production Engineering (DICMAPI) University of Naples Federico II, P.le Tecchio 80, 80125 Naples, Italy and CEINGE Advanced Biotechnologies, 80145 Naples, Italy. [4]Department of Experimental Medicine, University of Campania "Luigi Vanvitelli", via Luciano Armanni 5, 80138, Naples, Italy. [*]Correspondence and requests for materials should be addressed to Giovanna Cacciapuoti (email:giovanna.cacciapuoti@unicampania.it).

Analysis of the contributions of doubling and migration times

The wound healing can be described as depending on the contribution of two different cell processes: cell motility and proliferation[1,2]. Cell motility can be modeled as a Fickian diffusion and depends on a random motility coefficient μ, that is analogous to a diffusion coefficient, while cell proliferation can be modeled as a logistic growth, and depends on k, a parameter analogous to the kinetic constant of a chemical reaction which can be estimated as the reciprocal of the cell doubling time ($k = ln(2)/\, t_D$), approximating the proliferation rate as a simple first order kinetic. An estimate of which one is the dominant process between cell motility and proliferation, can be obtained by calculating the Thiele modulus (Φ), defined as the ratio between the characteristic time of the migration and proliferation processes[2,3].

$$\Phi = \frac{t_\mu}{t_D} = \frac{b_0}{2}\sqrt{\frac{k}{\mu}} \quad \text{Equation (1)}$$

In this equation, b_0 is the distance of the two edges of the wound at time 0, in μm, representing the characteristic length along which cell diffusion occurs, in our case b_0 is typically about 800 μm. In the case of high values of Φ, ($t_\mu > t_D$), the duplication is the dominant process, being faster respect to motility.

The cell front propagates at a constant velocity, perpendicularly to the wound edge, reducing the size of the wound area[4]. Consequently, this wound closure velocity is directly related to the propagating cell front velocity (v, μm/h) i.e. $v = \alpha \cdot \frac{b_0}{2}$. The propagation cell front velocity is related to the values of the random motility coefficient and cell doubling time:

$$v = \sqrt{\frac{4\, \mu \ln{(2)}}{\tau_D}} \quad \text{Equation (2)}$$

The values of the doubling time of the control cells were those reported in ATCC data sheets i.e. 38 h and 47 h for MDA-MB-231 and MDA-MB-468, respectively, while the doubling time of treated cells was estimated by direct counting of mitosis events and cell number during time lapse experiments assuming that the ratio between the number of mitoses and the number of cells, M, is inversely proportional to the doubling time. The doubling times corresponding to different cell treatments were estimated according to the proportion:

$$\frac{M_{CTR}}{M_{\text{treatment}}} \propto \frac{t_{D,\text{treatment}}}{t_{D,CTR}} \rightarrow t_{D,\text{treatment}} \approx \frac{M_{CTR}}{M_{\text{treatment}}} \cdot t_{D,CTR}.$$

Once obtained the measurement of the wound closure velocity (α) from the analysis of the time lapse experiments and the doubling time (t_D) for each experimental condition, it is possible to calculate the random motility coefficient μ from reverse Equation (2) and consequently the Thiele modulus (1).

According to our estimates the doubling time (t_D) of MDA-MB-231 cells, was drastically increased by APE being about doubled in treated sample (~ 76 h) compared to the control, with a limited dependence on APE concentration. The migration time t_μ calculated from the wound closure velocity measurement was also increased, with a not negligible dependency on APE concentration resulting about 2-fold and 5-fold higher than control in cells treated with APE 200 and 300 µM EqC, respectively (**Fig. S2, left**). Data are statistically significant, as verified by *P* values. The comparison of the values of Thiele modulus estimated from Equation (1) for the different treatments (data not reported for the sake of brevity), showed a limited influence of APE respect to control (Φ is order 1) indicating that cell motility can be considered the fast, but only marginally the leading mechanism. Overall, considering the balance between the measured increment in migration and duplication times under the experimental conditions we investigated, it is possible to conclude that APE affected both cell motility and proliferation, resulting in an overall significant reduction in the wound closure efficiency.

The ability of NAC and JNK to revert the effects of APE on cell migration was confirmed by the evaluation of the doubling time that was drastically reduced, with values of 50 h and 38 h in cells treated with APE 200 µM after pretreatment with SP600125 and NAC, respectively. The migration times calculated in the same experimental conditions showed values comparable to that of control (**Fig. S2, right**). Notably, we found that the Thiele modulus in treated samples was comparable to the control, remaining of order 1, indicating that motility and proliferation are both relevant to the migration process. It is worth mentioning this is not always the case, being reported in the literature examples of systems with $\Phi \gtreqless 1$, suggesting the presence of a leading mechanism[2].

References

1. Ascione, F. *et al.* Comparison between fibroblast wound healing and cell random migration assays in vitro. *Exp Cell Res*. **347**, 123-132 (2016).
2. Ascione, F., Caserta, S. & Guido, S. The wound healing assay revisited: A transport phenomena approach. *Chem Eng Sci.* **160**, 200-209 (2017).
3. Ascione, F., Guarino, A.M., Calabrò, V., Guido, S. & Caserta, S. A novel approach to quantify the wound closure dynamic, *Exp Cell Res*. **352**, 175-183 (2017).
4. Maini, P.K., McElwain, D.L.S. & Leavesley, D.I. Traveling wave model to interpret a wound-healing cell migration assay for human peritoneal mesothelial cells. *Tissue Eng.* **10**, 475-482 (2004).

Supplementary Figures

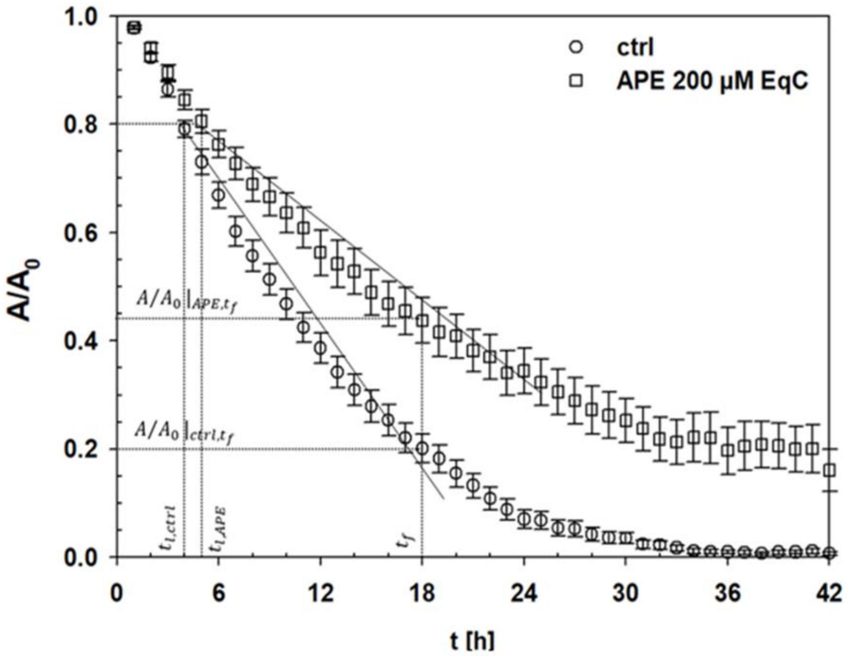

Figure S1. Graphical analysis of the parameters of a typical A/A_0 vs t curve in control and APE-treated cells. We consider as an example two curves relative to control and APE 200 µM EqC treatment. The wound closure velocity of each experimental condition was assessed as the slope of the curve between the lag time (t_l) and the time at which A/A_0 anticipates by 0.1 the *plateau* value (0 in the case of control). Considering that A/A_0 linearly decreases with the time, $A/A_{0_i} = 1 - \alpha_i \cdot t$, with i = ctr and/or APE 200 µM EqC, we obtained the values of the wound closure velocities α_{ctrl} and α_{APE} for control and APE-treated sample, respectively. The $\left.\frac{A}{A_0}\right|_{t_f}$ values corresponding to the wound area A/A_0 at the time (t_f), i.e. the time when the A/A_0 for control is 20%, are also shown.

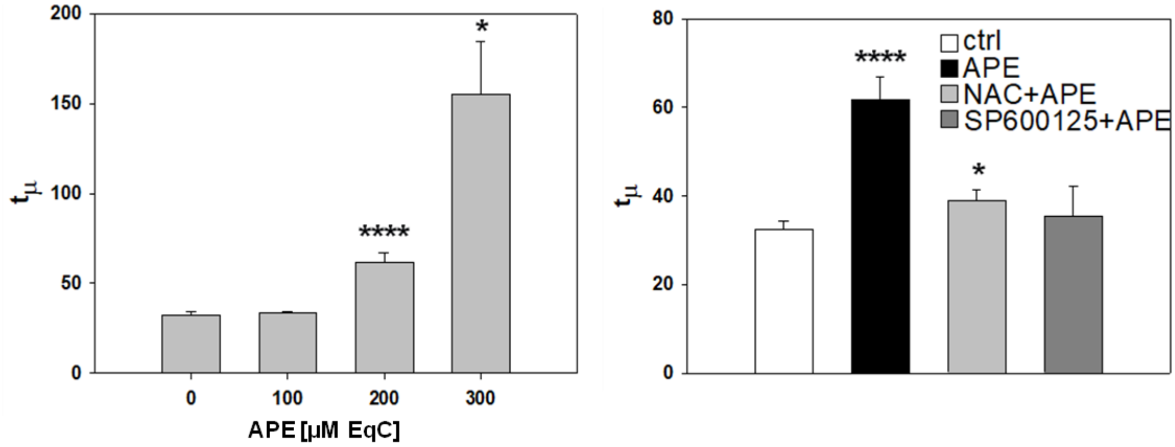

Figure. S2. **Effect of APE, NAC, and SP600125 on migration time.** Values of migration time t_μ were calculated according to the Fisher-Kolmogoroff equation. Bar diagrams show the t_μ values of cells treated or not with different APE concentrations (**left**) and with 200 μM EqC APE with or without 1 h pretreatment with 5 mM NAC and 5 μM SP600125 (**right**). Data reported in the histogram are the mean of several independent fields of view. Standard error of the mean was calculated and the t-test was performed to verify the statistical significance of the differences among the control and treated samples. (*$P < 0.05$, ****$P < 5 \cdot 10^{-5}$).

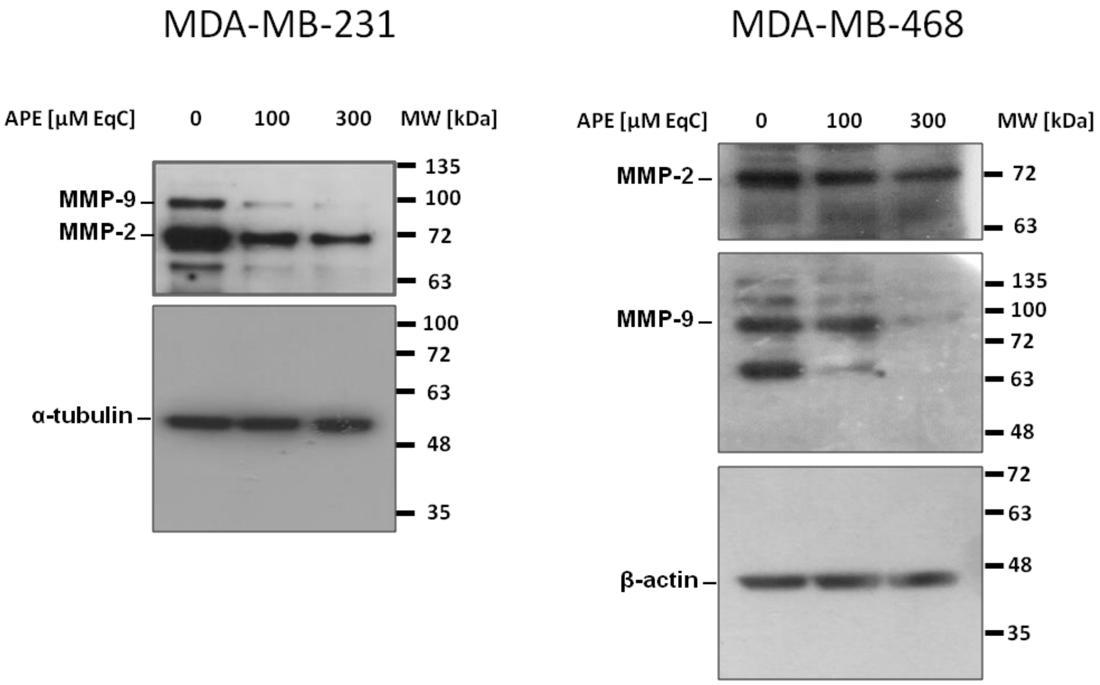

Figure S3. APE inhibited cell growth and migration of MDA-MB-231 and MDA-MB-468 cells. The cropped blots are used in the main figure (Figure 1d).

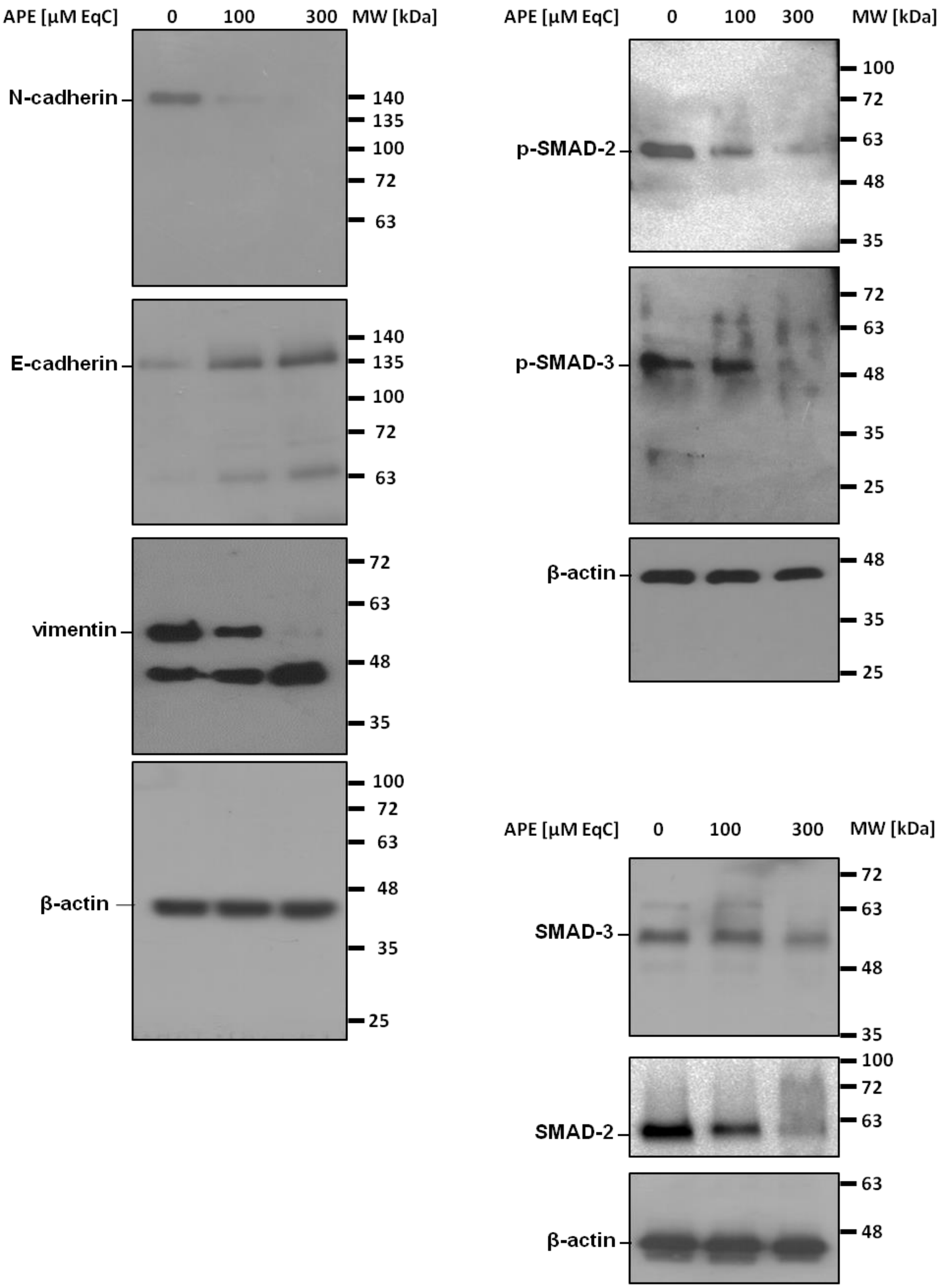

Figure S4. Effect of APE on the levels of EMT-related proteins in MDA-MB-231 cells. The cropped blots are used in the main figure (Figure 2a).

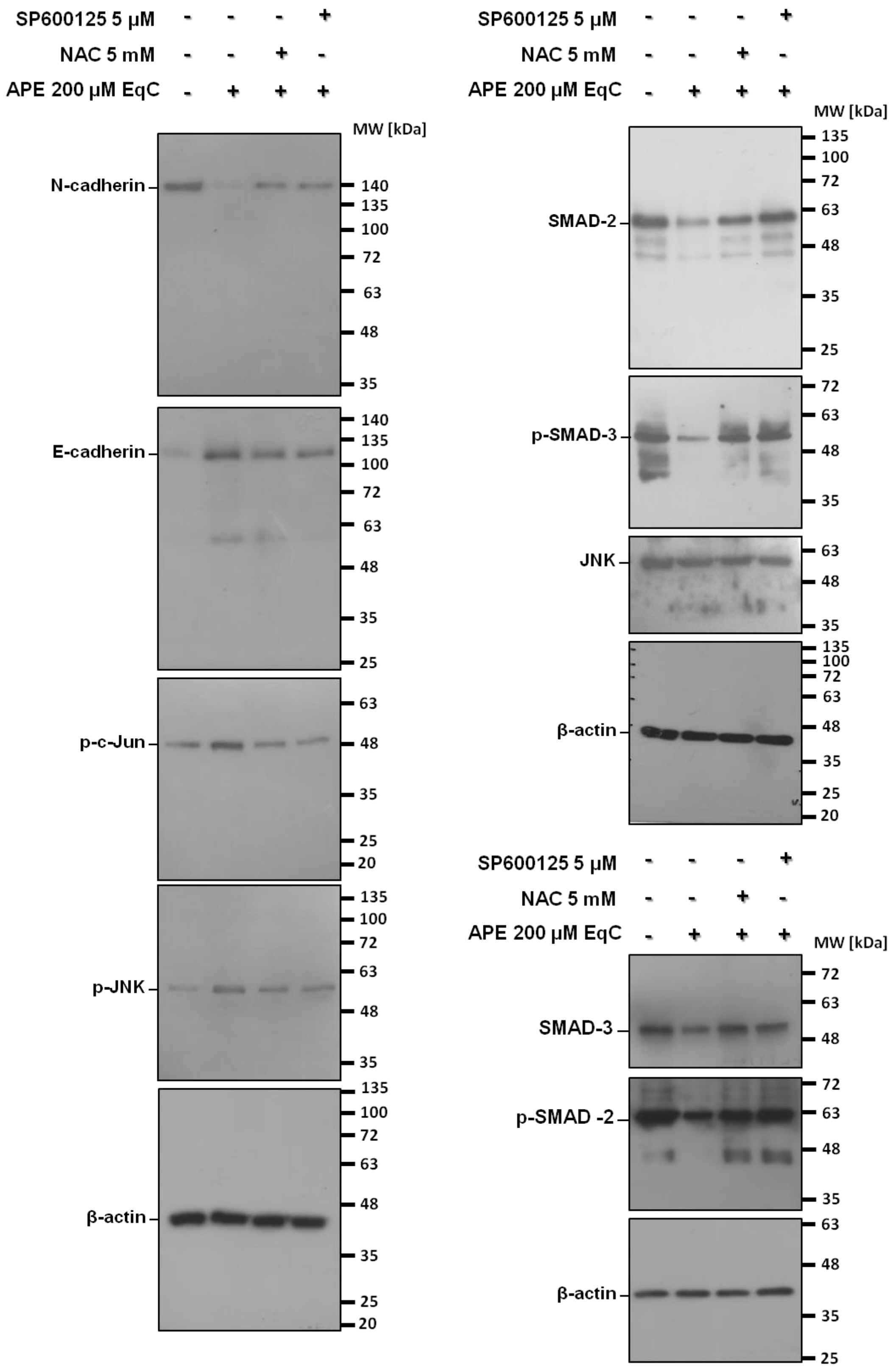

Figure S5. APE inhibited MDA-MB-231 cell migration through ROS/JNK signaling activation. The cropped blots are used in the main figure (Figures 3c). **ROS/JNK signaling activation mediated APE-induced switch from N- to E-cadherin expression.** The cropped blots are used in the main figure (Figure 4a).

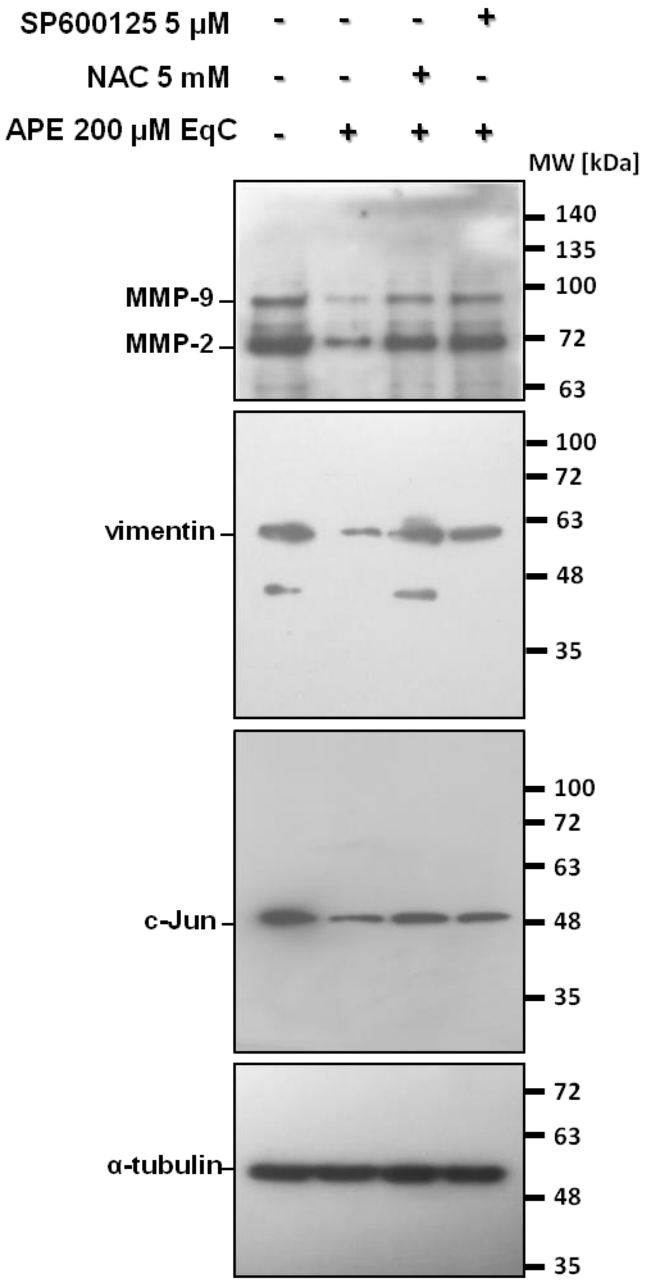

Figure S6. APE inhibited MDA-MB-231 cell migration through ROS/JNK signaling activation. The cropped blots are used in the main figure (Figure 3c). **ROS/JNK signaling activation mediated APE-induced vimentin downregulation and vimentin network organization.** The cropped blots are used in the main figure (Figure 5b).

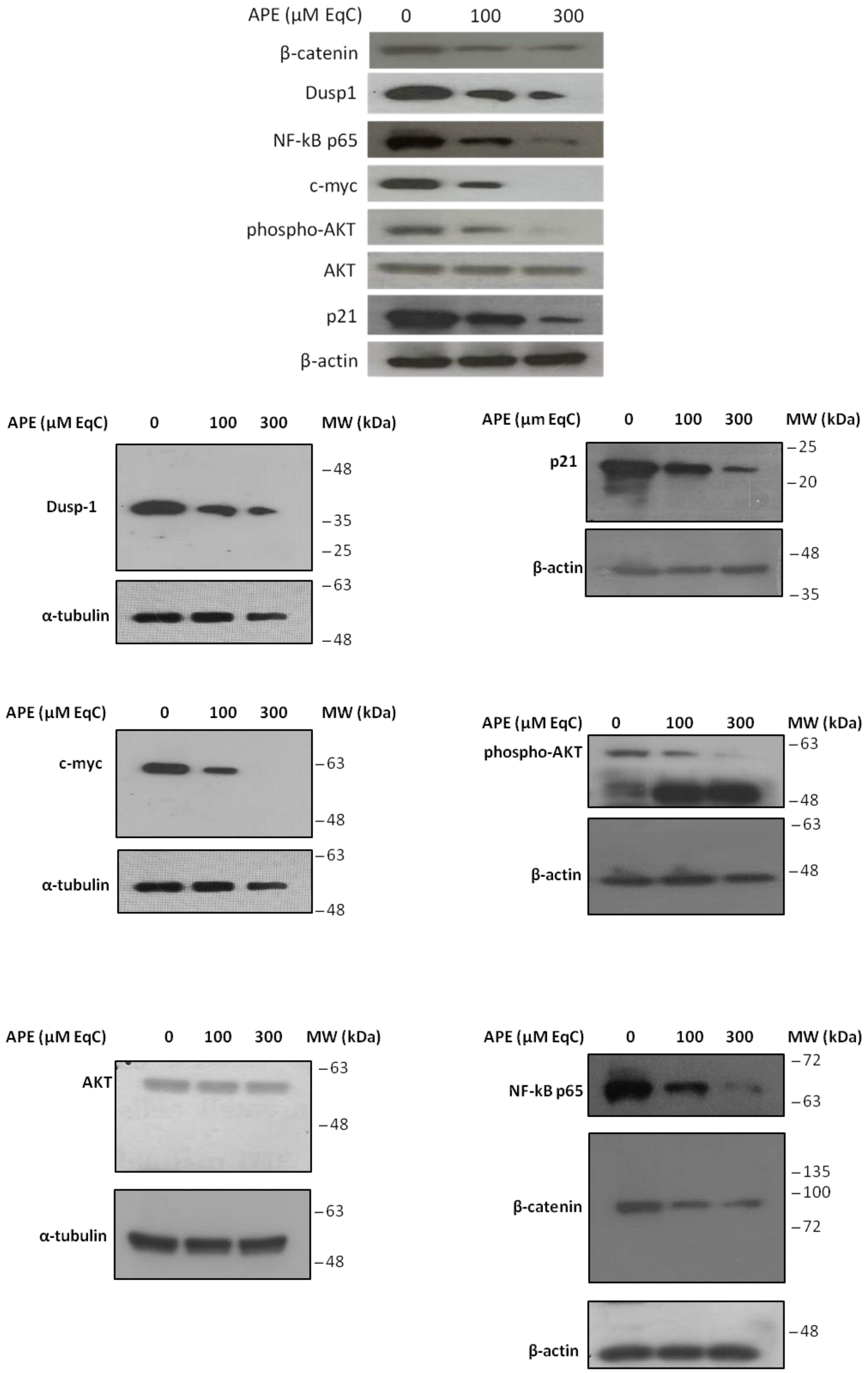

Figure S7. APE downregulates AKT, p21, NF-kB, c-myc, β-catenin, and Dusp-1. Cropped and uncropped blots have been presented as original results in *Sci Rep.* 9, 13045 (2019).

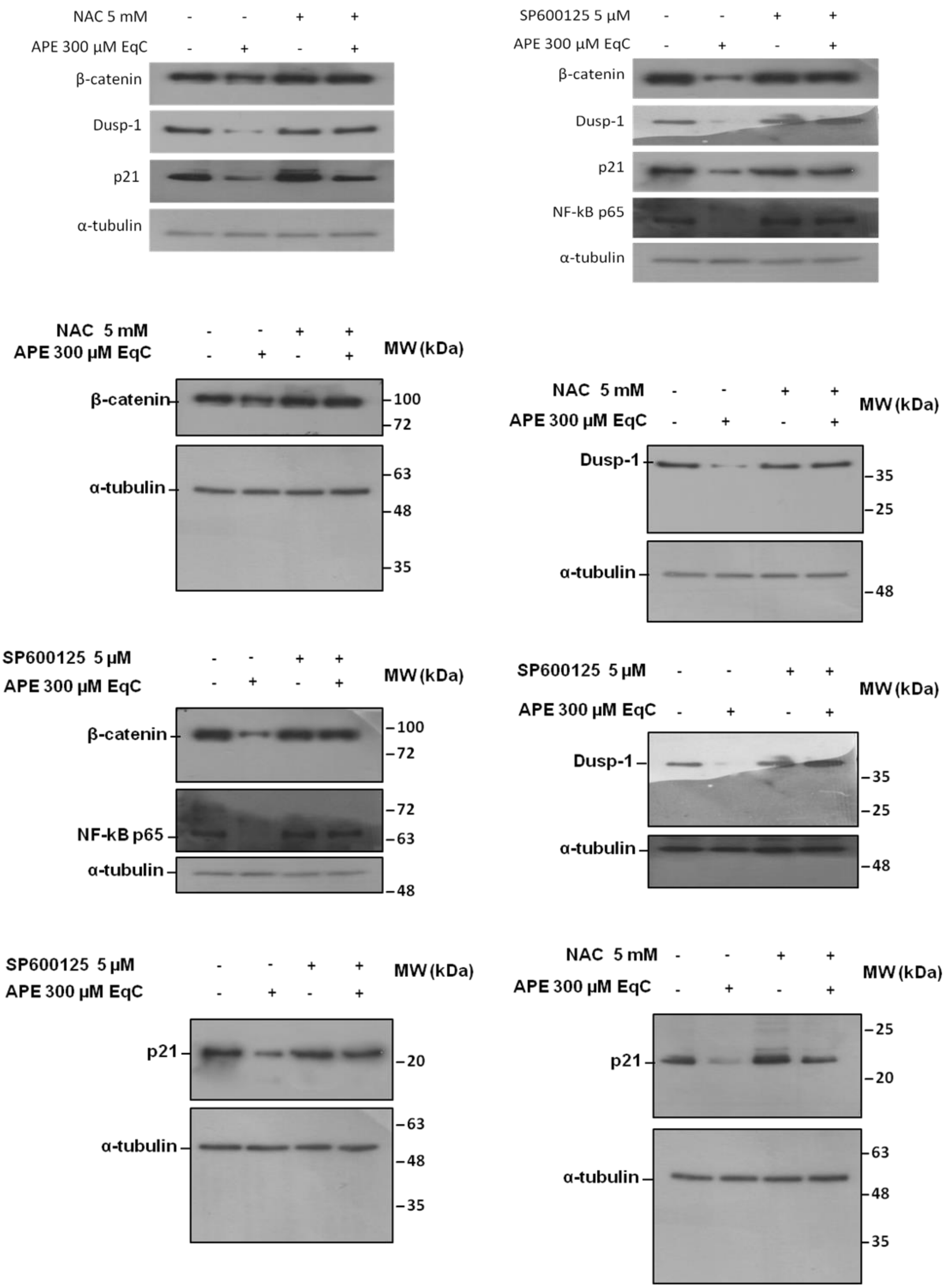

Figure S8. Effect of NAC and SP600125 on APE-induced inhibition of p21, NF-kB, β-catenin and DUSP-1. Cropped and uncropped blots have been presented as original results in *Sci Rep.* 9, 13045 (2019).

Supplementary Videos

video S1. Time-lapse video microscopy of MDA-MB-231 cells control.

video S2. Time-lapse video microscopy of MDA-MB-231 treated with 100 μM EqC APE.

video S3. Time-lapse video microscopy of MDA-MB-231 treated with 200 μM EqC APE.

video S4. Time-lapse video microscopy of MDA-MB-231 treated with 300 μM EqC APE.

video S5. Time-lapse video microscopy of MDA-MB-468 control.

video S6. Time-lapse video microscopy of MDA-MB-468 treated with 100 μM EqC APE.

video S7. Time-lapse video microscopy of MDA-MB-468 treated with 200 μM EqC APE.

video S8. Time-lapse video microscopy of MDA-MB-468 treated with 300 μM EqC APE.

video S9. Time-lapse video microscopy of MDA-MB-231 control.

video S10. Time-lapse video microscopy of MDA-MB-231 treated with 200 μM EqC APE.

video S11. Time-lapse video microscopy of MDA-MB-231 treated with 200 μM EqC APE after pretreatment with 5 mM NAC.

video S12. Time-lapse video microscopy of MDA-MB-231 treated with 200 μM EqC APE after pretreatment with 5 μM SP600125.
